# A sensitive assay for dNTPs based on long synthetic oligonucleotides, EvaGreen dye and inhibitor-resistant high-fidelity DNA polymerase

**DOI:** 10.1093/nar/gkaa516

**Published:** 2020-06-23

**Authors:** Janne Purhonen, Rishi Banerjee, Allison E McDonald, Vineta Fellman, Jukka Kallijärvi

**Affiliations:** Folkhälsan Research Center, Helsinki, Finland; Stem Cells and Metabolism Research Program, Faculty of Medicine, University of Helsinki, Finland; Folkhälsan Research Center, Helsinki, Finland; Stem Cells and Metabolism Research Program, Faculty of Medicine, University of Helsinki, Finland; Department of Biology, Wilfrid Laurier University, ON, Canada; Folkhälsan Research Center, Helsinki, Finland; Stem Cells and Metabolism Research Program, Faculty of Medicine, University of Helsinki, Finland; Department of Clinical Sciences, Lund, Pediatrics, Lund University, Sweden; Children's Hospital, Helsinki University Hospital, Finland; Folkhälsan Research Center, Helsinki, Finland; Stem Cells and Metabolism Research Program, Faculty of Medicine, University of Helsinki, Finland

## Abstract

Deoxyribonucleoside triphosphates (dNTPs) are vital for the biosynthesis and repair of DNA. Their cellular concentration peaks during the S phase of the cell cycle. In non-proliferating cells, dNTP concentrations are low, making their reliable quantification from tissue samples of heterogeneous cellular composition challenging. Partly because of this, the current knowledge related to the regulation of and disturbances in cellular dNTP concentrations derive mostly from cell culture experiments with little corroboration at the tissue or organismal level. Here, we fill the methodological gap by presenting a simple non-radioactive microplate assay for the quantification of dNTPs with a minimum requirement of 4–12 mg of biopsy material. In contrast to published assays, this assay is based on long synthetic single-stranded DNA templates (50–200 nucleotides), an inhibitor-resistant high-fidelity DNA polymerase, and the double-stranded-DNA-binding EvaGreen dye. The assay quantified reliably less than 50 fmol of each of the four dNTPs and discriminated well against ribonucleotides. Additionally, thermostable RNAse HII-mediated nicking of the reaction products and a subsequent shift in their melting temperature allowed near-complete elimination of the interfering ribonucleotide signal, if present. Importantly, the assay allowed measurement of minute dNTP concentrations in mouse liver, heart and skeletal muscle.

## INTRODUCTION

Deoxyribonucleoside triphosphates (dNTPs) are building blocks of DNA and, accordingly, their cellular concentration is highest during active DNA synthesis, such as in proliferating cells (1). Basal dNTP levels in non-proliferating or postmitotic cells are only ∼5% that of in cells in S phase and are mainly required for DNA repair and maintenance of mitochondrial DNA (1,2). While studying pyrimidine biosynthesis in a mouse model of mitochondrial respiratory complex III deficiency (3), we encountered the problem that no suitable method, applicable for the general laboratory without special instrumentation, to measure dNTPs from tissue samples exists. Traditional enzymatic methods to measure dNTPs have relied on the incorporation of radioactive dNTPs to a complementary strand of a DNA template by a DNA polymerase (4,5). These methods have mainly been used to measure dNTPs from actively proliferating cultured cells with high dNTP concentrations and a limited complexity of interfering biological matrix. Recently, a HPLC-MS-based method was developed that allows quantification of tissue dNTP levels from a feasibly small tissue amount (15–30 mg) (6). However, not every laboratory has suitable HPLC–MS equipment and expertise. A simple probe hydrolysis-based enzymatic fluorometric assay has also been developed to measure dNTPs from cultured cells (7). This assay utilizes synthetic DNA templates with a 3′ primer-binding region, mid-template dNTP-detection region, 5′ probe-binding sequence, and a Taq DNA polymerase with 3′ to 5′ exonuclease activity to hydrolyse the probe - an adaptation of typical TaqMan qPCR chemistry. The authors achieved low baseline fluorescence by using dual-quenched 6-carboxyfluorescein (FAM) -labelled probes. Due to the simplicity of this fluorometric assay, we set out to test it as a means to quantify dNTPs in mouse liver extracts. We found some limitations in the sensitivity and robustness of this method, especially concerning complex tissue extracts with low dNTP concentrations. Therefore, we developed a novel and even simpler assay based on a long synthetic template, EvaGreen DNA dye, and a robust inhibitor-resistant high-fidelity DNA polymerase.

## MATERIALS AND METHODS

### Reagents

Integrated DNA Technologies (IDT) (Coralville, Iowa, USA) synthetized all DNA oligonucleotides used for the dNTP measurements (Supplementary Table S1). The IDT Ultramer technology was applied for the synthesis of the 197 nucleotide (nt) templates which were then desalted. The primers and the 50-nt templates for the novel assay were PAGE purified as were the templates for the published (7) probe-hydrolysis based assay. The probes were HPLC purified. Supplementary Table S2 lists all critical reagents, materials and instrumentation of the study.

### Cell culture

Mouse hepatoma cells (Hepa1–6), mouse embryonic skin fibroblasts (NIH/3T3), human neonatal skin fibroblasts (8), and monkey fibroblast-like kidney cells (COS-1) were cultured in standard high-glucose medium (DMEM, 10% fetal bovine serum, Glutamax, penicillin and streptomycin). Human colorectal cancer cells (HCT116) were cultured similarly but RPMI1640 containing 25 mM NaHCO_3_ was used as a base medium.

For the dNTPs measurements, the cells were plated into 75- or 25-cm^2^ flasks (ThermoFisher Scientific) and allowed to reach the logarithmic growth phase. Hepa1–6 cells were treated with either vehicle or 5 μM 5-fluorouracil (from 5 mM stock in H_2_O) or 200 nM myxothiazol (from 0.5 mM stock in EtOH) for ∼6 h. For sample collection, the cells were washed with phosphate-buffered saline (PBS) and detached with TrypLE (ThermoFisher Scientific). Then, 3.5–10 ml ice-cold PBS was added, an aliquot was saved for counting of the cells, and the cells were pelleted by centrifugation (300g for 5 min at +4°C). The cell pellets were resuspended in 550 μl of ice-cold 60% MeOH and stored at –80°C until extraction.

### Generation of a Pacific oyster alternative oxidase-expressing mammalian cell line

Alternative oxidase (AOX) cDNA from Pacific oyster (*Crassostrea gigas*) (Genbank, #ACL31211) (9) was cloned into the pcDNA6A mammalian expression vector (Invitrogen). First, an internal EcoRI site was removed by PCR mutagenesis (primers 5′-ATT CGC ACC AGC AAT GGG CT-3′ and 5′-ACC ACT GAC CCT CAG TAG AAT T-3′) and then the coding sequence was PCR-amplified (primers 5′-ATGAATTC ATG GGA AGT TTG CGA CAA ATA AC-3′and 5′-ATCTCGAG TCA CTT CCC TGG CTC ATA AGG-3′ or 5′-ATCTCGAG CTT CCC TGG CTC ATA AGG ATT-3′) and cloned into pcDNA6A with or without a C-terminal V5–6XHis tag. The cDNA was further subcloned into the Sleeping Beauty transposon (10) donor plasmid ITR\CAG-MCS-IRES-Puro2A-Thy1.1\ITR (a kind gift from Dr Madis Jakobson, Max Planck Institute of Biochemistry, Germany).

The expression and subcellular localization of the Pacific Oyster AOX (PoAOX) was confirmed by transfecting the pcDNA6-poAOX-V5–6XHis construct into COS-1 cells, followed by immunofluorescence staining (Supplementary Figure S1A). To visualize mitochondria and PoAOX, mouse anti-MT-CO1 (ab14705, Abcam) and rabbit anti-V5 (AB3792, Merck) antibodies were used, respectively.

To generate a cell line stably expressing PoAOX, Hepa1–6 cells were co-transfected with a plasmid encoding SB100X transposase and the Sleeping Beauty transposon donor plasmid carrying the PoAOX-V5–6XHis insert (1:10 ratio) using FuGENE® HD transfection reagent. For a control cell line, the empty ITR\CAG-MCS-IRES-Puro2A-Thy1.1\ITR plasmid was transfected. After puromycin selection (2 μg/ml), resistant colonies were pooled, and after five passages the cells were cultured without puromycin. The activation and activity of poAOX was assessed by measuring antimycin A-resistant respiration using Oxygraph-2k (OROBOROS instruments) (Supplementary Figure S1B).

### Collection and homogenization of mouse tissues

Mice of congenic C57BL/6JCrl background were maintained in individually ventilated cages at the animal facilities of University of Helsinki, Finland, under the internal license KEK19–033. Male mice (110–140 days old) were euthanized by cervical dislocation and the tissues were immediately excised and placed in liquid nitrogen and stored at –80°C. The frozen tissue samples were directly homogenized in ice-cold 60% MeOH (20–40 mg/0.55 ml) using battery-operated microtube pestles for liver and roughened glass-to-glass tissue grinders for heart and skeletal muscle (whole calf).

### Extraction of dNTPs

The cultured cells and mouse tissue homogenates in 60% MeOH were further denatured by incubation at 95°C for 3 min to quench residual enzymatic activity and to facilitate the extraction. Supernatants (550 μl) from an 18500g centrifugation (6 min at +4°C) were run through 3-kDa cut-off centrifugal filters (Amicon Ultra-0.5 ml, Merck) into pre-weighted collection tubes to remove remaining macromolecules. Next, MeOH and hydrophobic metabolites were removed by washing the extracts twice with 1.4 ml diethyl ether. Residual diethyl ether was evaporated using Speed-Vac Plus SC110A centrifugal vacuum evaporator (Savant Instruments, Farmingdale, NY, USA) (typically 15 min at 65°C). The remaining liquid after evaporation was estimated by weighing the tubes and assuming that 1 μl weights 1 mg. Supplementary Table S3 lists the extract dilution used for the different measurements from cultured cells. The tissue extract dilutions are given in the figures, and tables, and their respective legends. For initial test runs (Supplementary Figure S2A–D), we also followed a published extraction protocol without modifications (7). The extracts were stored at –80°C until use.

### Probe hydrolysis-based enzymatic assay for dNTPs

For the fluorochrome-quencher-probe -based polymerase assay for dNTPs, we followed the published protocol (7) with minor modifications. To accommodate the relatively higher sample volume and 384-well format, we lowered the reaction volume from 25 to 10 μl and added the reaction components as a 2×-master mix followed by addition of an equal volume of sample. The final reaction concentrations were: 0.4 μM primers, probes and templates, 100 μM non-limiting dNTPs, 2 mM MgCl_2,_ and AmpliTaq Gold DNA polymerase at 35 U/ml. The reaction was started by activating the hot-start polymerase by a 10-minute incubation step at 95°C. Baseline fluorescence was read at 60°C immediately after polymerase activation. Thereafter, the reaction was allowed to proceed at 60°C while monitoring the fluorescence. The Bio-Rad CFX384 qPCR instrument served as a thermal cycler and fluorometer for the assay.

### Q5 DNA polymerase and EvaGreen-based assay for dNTPs using 197-nt templates

The following reaction concentrations were found to be optimal: 1× Q5 reaction buffer: 0.275 μM primer and 0.25 μM template, 50 μM non-limiting dNTPs and 1.25 μM EvaGreen (Biotum). The optimal Q5 DNA polymerase (New England Biolabs) amount was template-dependent: 20 U/ml for dATP and dTTP, and 10 U/ml for dGTP and dCTP detection. The reaction components were prepared as a 2×-master mix and 5 μl of the mix was pipetted into opaque wells of 384-well PCR-plate (Bio-Rad). Then an equal volume of sample was added. The reaction set-up was prepared on ice. For initial optimization runs, the thermal cycler (CFX384, BioRad) was programmed to heat the plate to 98°C (10s) followed by cooling to the final reaction temperature (66°C was considered optimal). The baseline fluorescence was immediately read after reaching the target temperature. Thereafter, the fluorescence (SYBR Green/FAM channel of the instrument) was recorded typically once every 5 min for 75 min. Later, the assay was found more sensitive when the baseline and the end-point fluorescence were read at a temperature above the primer annealing temperature (75°C). With this approach we found it best to limit the actual reaction time at 66°C to 55 min for dATP, 40 min for dTTP and dCTP, and 20 min for dGTP detection.

### Elimination of interfering signal from ribonucleotides

The reaction component concentrations were the same as for the basic assay, with the exception that the templates were shortened to 50 nt and 0.2 U/ml thermostable RNAse HII (IDT) was included in the reaction mixture. The following reaction times at 66°C were found optimal for the 50-nt templates: 50 min for dATP, 25 min for dTTP and dCTP, and 15 min for dGTP detection. The baseline and the end-point fluorescence were read above the melting temperature of RNAse HII-nicked DNA as determined according to melt curve analyses: 75°C for dATP and dCTP, 78°C for dTTP and 73.5°C for dGTP.

### Denaturing gel electrophoresis

The assay reactions of 20 μl volume with and without 0.2 U/ml thermostable RNAse HII were stopped by decreasing the temperature to 0°C and by addition of 12 mM EDTA. Then the reactions were dried using Speed-Vac Plus SC110A centrifugal vacuum evaporator and dissolved in 10 μl of 8 M urea and 6% Ficoll in TBE (89 mM Tris-borate, 2 mM EDTA, pH 8.3). After 2 min denaturation at 95°C and snap-cooling on ice, the samples were resolved in pre-heated 13% polyacrylamide 8 M urea–TBE gels. The single-stranded DNAs (ssDNAs) were visualized with PAGE GelRed stain (Biotium). Modifications for the post-reaction alkaline cleavage of ribonucleotide bonds are explained in the supplementary figure legends.

### Data analysis

To export the raw fluorescence values for data analysis, the automatic baseline correction by the qPCR instrument was turned off. Nonlinear least squares regression curves were calculated using GraphPad Prism 8.2.1 (GraphPad Software, CA, USA). Signal-to-noise ratio was defined as background-subtracted fluorescence (*n* = 3) divided by the standard deviation of the background (*n* = 6). The lowest limit of quantification (LLOQ) was estimated as the lowest concentration at which 33% differences could still be reliably distinguished. At this concentration signal-to-noise ratio was equal to or higher than 10. The measured concentrations were corrected for losses during 3 kDa cut-off filtration. This was accomplished by weighing the residual liquid in the filter device and assuming a 0.92 g/ml density of 60% MeOH at 0°C.

### Step-by-step protocol

A detailed step-by-step protocol for sample extraction and measurement procedure is provided as a Supplementary file.

## RESULTS

### Design of the assay concept

**Figure 1.**
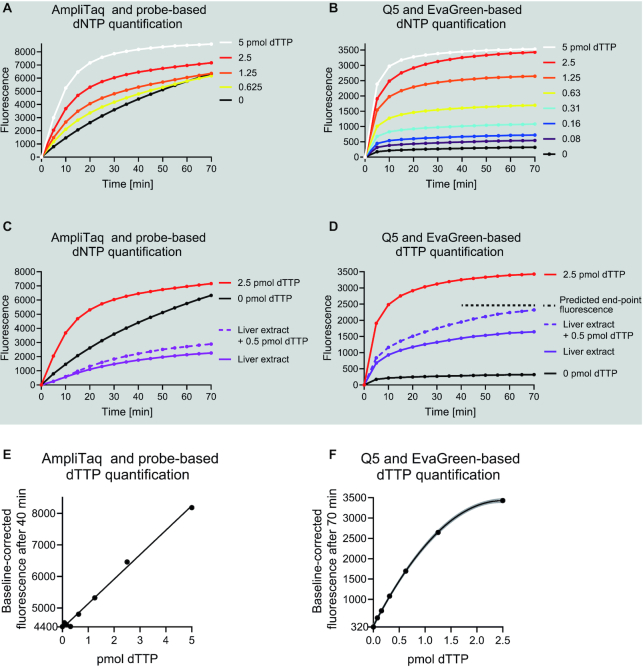
Long synthetic DNA oligonucleotides, an inhibitor-resistant high-fidelity DNA polymerase and EvaGreen detection chemistry allow quantification of dNTPs from mouse liver extracts. (**A**) A representative quantification of dTTP using a published fluorometric probe-hydrolysis-based assay. For clarity, curves from three lowest standard samples (0.31–0.08 pmol) are not shown. (**B**) A representative quantification of dTTP using Q5 DNA polymerase, 197-nt template and EvaGreen detection chemistry. (**C**, **D**) dTTP signal generated by the fluorometric methods from a high concentration mouse liver extract with and without 0.5 pmol dTTP spike-in calibrant. The extract volume was adjusted to 2 μl per mg of initial tissue weight. The extract comprised half of the reaction. (**E**, **F**) Standard curves generated from the end-point baseline-corrected fluorescence values. The lowest y-axis value shows the background signal. Gray lines present the 95% confidence interval of a polynomial curve fit.

**Figure 2.**
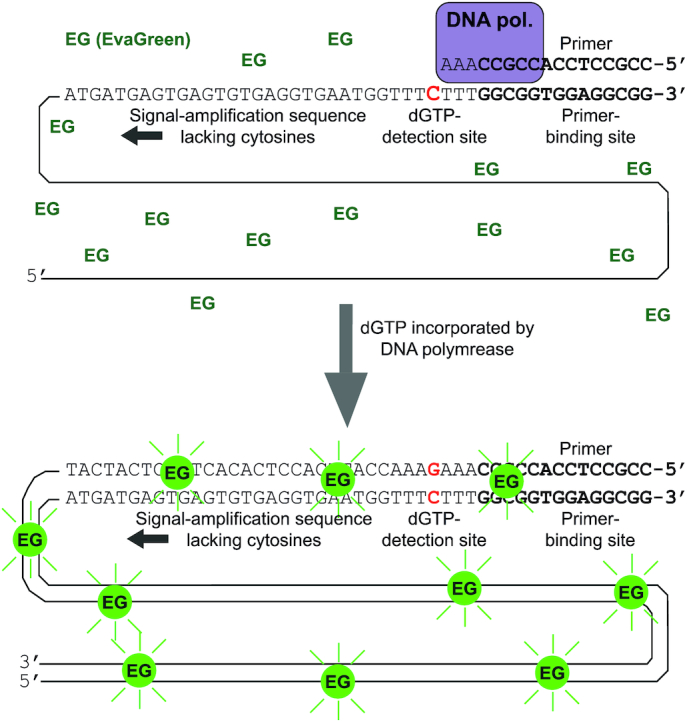
The principle of the novel dNTP assay. Single-stranded DNA templates comprise a 3′ primer-binding site followed by a single detection site for the dNTP to be quantified (here dGTP). The rest of the template, in the 5′ direction, does not contain any cytosines, and its sole purpose is to maximize the amount of double-stranded DNA synthesized per a single dGTP incorporated by the DNA polymerase. The double-stranded DNA binds the EvaGreen fluorochrome and renders it fluorescent, which is measured. In this method, the template length determines the signal amplification.

We initially tested the published probe hydrolysis-based enzymatic assay (7) for the measurement of hepatic dNTPs. However, we encountered two problems. First, the suggested AmpliTaq Gold DNA polymerase appeared to be sensitive to inhibition by liver, heart and skeletal muscle extracts, as shown by signal below background level (Figure 1 and Supplementary Figure S2A–E). To dissect the cause of the problem, we employed cultured cancer cells (HCT116) with much higher dNTP concentrations. Dilute extracts from HCT116 cells did not cause as obvious inhibition, but still we were only able to measure the most abundant dNTP, dTTP, without a trouble (Supplementary Figure S2E). Concentrating the cell extracts for the measurement of the less abundant dNTPs resulted in interference similar to the tissue extracts (Supplementary Figure S2F–H). Secondly, we observed a time-dependent increase in the background fluorescence, which greatly limited the sensitivity of the probe-based assay (Figure 1A, Supplementary Figure S2). We reasoned that the increasing background fluorescence could originate from the low-fidelity nature of the non-proofreading polymerase leading to eventual incorporation of a wrong (non-complementary) deoxyribonucletide in the presence of the highly unbalanced dNTP concentrations required by the assay. Given that no commercially available true high-fidelity polymerase with 5′ to 3′ exonuclease activity required for probe hydrolysis exists, we replaced the fluorochrome- and quencher-labelled probes with the EvaGreen double-stranded DNA (dsDNA) dye, increased the template length to 197 nt, and performed the assay with an inhibitor-resistant high-fidelity Q5 DNA polymerase (Figures 1B, D and 2). The designed template DNA oligomers had the same primer-binding and a single dNTP-detection site at the 3′end as in the original probe-based assay (Supplementary Table S1). The EvaGreen-based detection system produced an increasing fluorescence signal, which was proportional to the added limiting dNTP (Figure 1B). Importantly, the background fluorescence remained nearly constant during the whole reaction time, and the highly concentrated liver extracts spiked with known amounts of dNTPs also produced the expected increase in signal (Figure 1D).

**Figure 3.**
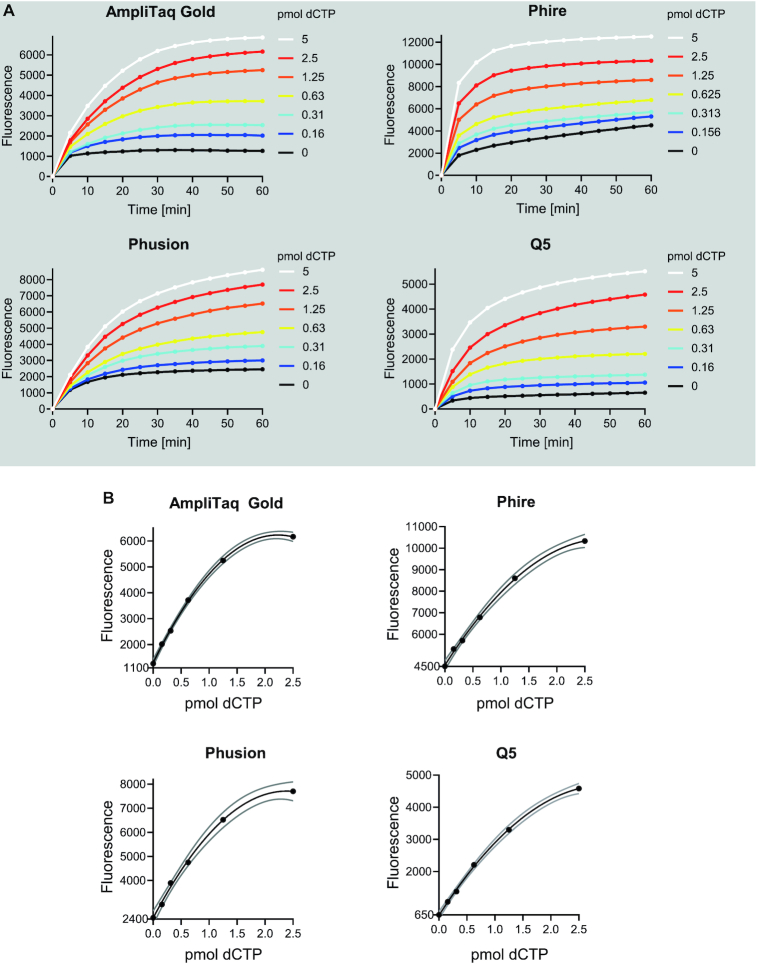
Assay kinetics with four different DNA polymerases. (**A**) Time-dependent increase in baseline-corrected fluorescence in the presence of different concentrations of limiting dNTP (dCTP). (**B**) Standard curves with second-order polynomial curve fit generated from end-point fluorescence values (1 h). Gray lines present the 95% confidence interval of the curve fit. Y-axis starts from the background signal. The same primer (0.4 μM), template (0.4 μM), non-limiting dNTP (100 μM) and EvaGreen (1×) concentrations were used for all reactions and in buffers supplied by the manufacturers of the DNA polymerases. Reaction temperatures were 60°C for AmpliTaq Gold (17.5 U/ml), 61.7°C for Phire (20 μl/ml) and Phusion (10 U/ml), and 68°C for Q5 (10 U/ml). The reaction temperature for Phire, Phusion and Q5 were chosen based on recommendations by the manufacturer. In the case of AmpliTaq Gold, the reaction temperature was selected based on the published probe-hydrolysis based assay.

To test our hypothesis that the error rate of the DNA polymerase could be a major determinant of the assay background, we performed the assay with EvaGreen detection using a typical Taq polymerase (AmpliTaq Gold), Phire DNA polymerase (2× fidelity versus Taq), Phusion polymerase (52× fidelity versus Taq) and Q5 polymerase (280× fidelity versus Taq). Indeed, the DNA polymerase with the highest rated fidelity produced the lowest background (Figure 3A). However, using the EvaGreen detection chemistry, the non-proofreading AmpliTaq Gold DNA polymerase also gave a rather stable background in comparison to the probe-based assay, suggesting that unspecific probe hydrolysis could also play a role in the probe-based detection. The novel EvaGreen-based assay also worked with Phire and Phusion DNA polymerases without any prior optimization (Figure 3A, B). The Phire DNA polymerase showed somewhat faster reaction kinetics than the other three enzymes. Based on the lowest background signal, and reported (11) inhibitor resistance in DNA extraction-free PCR, we chose to continue the optimization with Q5 DNA polymerase.

### Optimization of the reaction conditions

**Figure 4.**
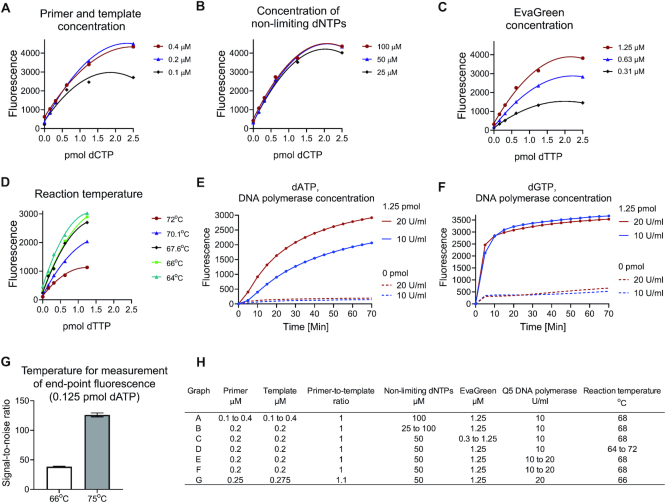
Optimization of EvaGreen- and Q5 DNA polymerase-based dNTP quantification. (**A–F**) Each critical reaction component and reaction temperature was varied, and the outcome evaluated. (**G**) The end-point fluorescence was measured at 66°C and after raising the temperature to 75°C to dissociate unused primers. (**H**) Assay conditions in graphs A to G. The standard curves were generated from the end-point baseline-corrected fluorescence values. The assessments were performed in triplicates. Supplementary Figure S3B shows the effect of increasing EvaGreen concentration above 1.25 μM. Supplementary Figure S3C-D shows the effect of different DNA polymerase concentrations on the dTTP and dCTP quantification.

We systematically optimized the concentration of key reaction components and temperature to maximize sensitivity and to decrease the cost of the assay (Figure 4). A decrease in primer and template concentration from 0.4 to 0.2 μM almost halved the background, without notable reduction in specific signal, for inputs <2.5 pmol (Figure 4A). A slight excess of primer (1.25-fold) over the template marginally improved the assay at the reaction temperature of 68°C (Supplementary Figure S3A). Non-limiting dNTP concentrations between 25 and 100 μM had little effect on the assay performance (Figure 4B). We chose the concentration of 50 μM as optimal for later runs The EvaGreen concentration recommended by the manufacturer for qPCR proved to be optimal (Figure 4C and Supplementary Figure S3B). The DNA polymerase concentration of 20 U/ml was required for the optimal quantification of dATP, whereas 10 U/ml was sufficient for dGTP quantification (Figure 4E-F). The polymerase concentration of 20 U/ml marginally improved the performance of dTTP and dCTP quantification in comparison to 10 U/ml (Supplementary Figure S3C-D). We varied the reaction temperature between 64 and 72°C and found that optima lied between 66 and 68°C (Figure 4D). Lower temperatures increased the background and higher ones compromised the assay. Because annealed primers bind EvaGreen (12), we also tested whether we could improve the signal-to-noise ratio by reading the baseline and the end-point fluorescence above the melting temperature of the primer extended to the detection site. Indeed, this approach increased the signal-to-noise ratio by approximately 3-fold (Figure 4G).

**Figure 5.**
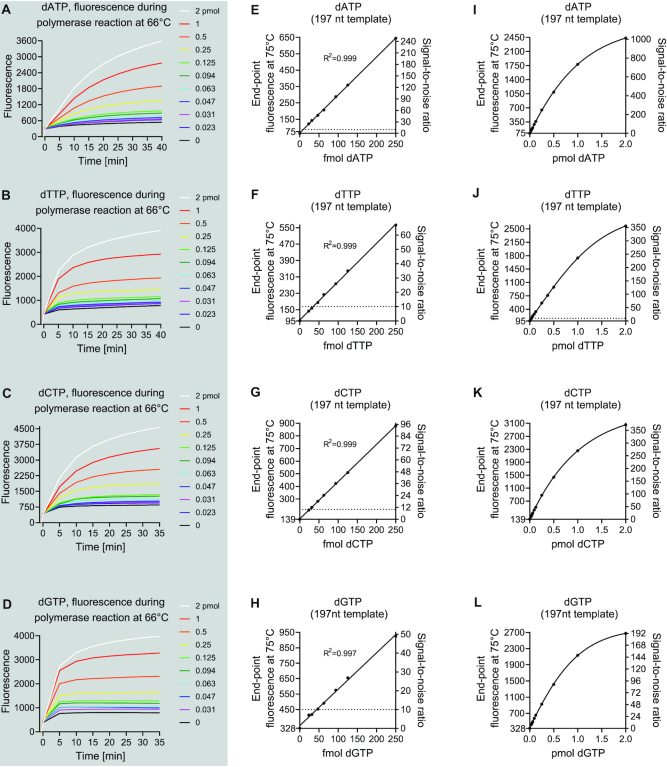
Assay performance with the 197-nt templates after optimization. (**A–D**) Time-dependent increase in baseline-subtracted fluorescence during the reaction phase of the assay. The baseline fluorescence was read at 75°C before progression to the primer annealing at 66°C and initiation of the reaction phase. (**E**–**L**) Standard curves generated from the end-point fluorescence values read after the increase of temperature to 75°C, a temperature at which the partially extended primers (17 nt) dissociate from templates. (E–G) Linear regression standard curves for the assay at low range. The dotted line shows signal-to-noise ratios of 10 as a reference to approximate the lowest limit of quantification. (I–L) Least-squares sigmoidal curve fits for the full range of the assay. The standard samples were assessed in triplicates and the background (0 pmol) in sextuplicates. The variation coefficients of replicates generally ranged from 1 to 6% and therefore error bars as standard deviation or standard error of mean are not show for clarity as they would not have been separable from the data point symbols. The left y-axis in the standard curve figures starts from the background value.

After all optimization, we found that the assay produced a linear response for inputs of 20–250 fmol (Figure 5). Sigmoidal curve fits, however, described the standard curves overall more accurately and gave a dynamic quantitative range up to 2 pmol (Figure 5I–L). We empirically verified the LLOQ down to 30 fmol for dATP, dTTP and dCTP. The detection of dGTP showed a slightly higher background, and therefore LLOQ of 47 fmol. The assay was the most sensitive for dATP and based on the signal-to-noise ratio of 10 as low as 9 fmol would be quantifiable. We also found that instead of baseline-subtracted end-point fluorescence, the standard curve could also be generated from end-point DNA melting temperature data by using the rate of fluorescence change at a specific temperature as a dependent variable (Supplementary Figure S4). This resulted in a further increase in signal-to-noise ratios.

**Figure 6.**
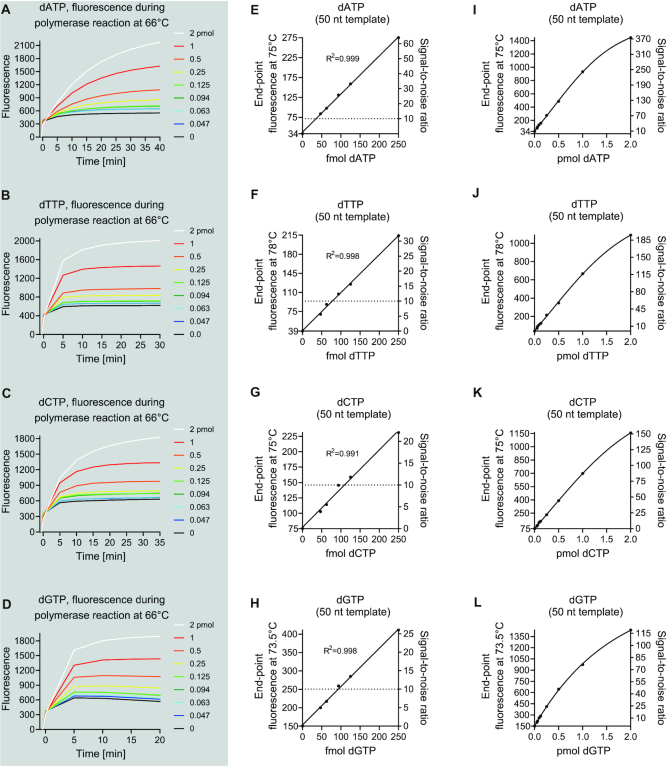
Assay performance with the 50-nt templates. (**A–D**) Time-dependent increase in baseline-subtracted fluorescence during the reaction phase of the assay. (**E**–**L**) Standard curves generated from the end-point fluorescence values measured at indicated temperatures (left y-axis title). (E–G) Linear regression standard curves for the assay at low range. The dotted line shows signal-to-noise ratios of 10 as a reference to approximate the lowest limit of quantification. (I–L) Least-squares sigmoidal curve fits for the full range of the assay. The standard samples were assessed in triplicates and the background (0 pmol) in sextuplicates. The variation coefficients of replicates generally ranged from 1 to 5% and therefore error bars as standard deviation or standard error of mean are not show for clarity as they would not have been separable from the data point symbols. The left y-axis in the standard curve figures starts from the background value.

As the high sensitivity of 197-nt templates is not required for all samples, we also tested the assay using 50-nt templates (Figure 6). As expected, the shorter templates decreased the fluorescence values and a large part of the signal came from the primer when the fluorescence was monitored during the reaction phase (Figure 6A–D). However, recording both the baseline and end-point fluorescence at a temperature above the primer annealing temperature diminished the primer-related signal. This allowed a usable quantitative range of 90 fmol to 2 pmol for all four dNTPs with the 50-nt templates (Figure 6E–L).

### Assessment of interference by ribonucleoside triphosphates (rNTPs)

**Figure 7.**
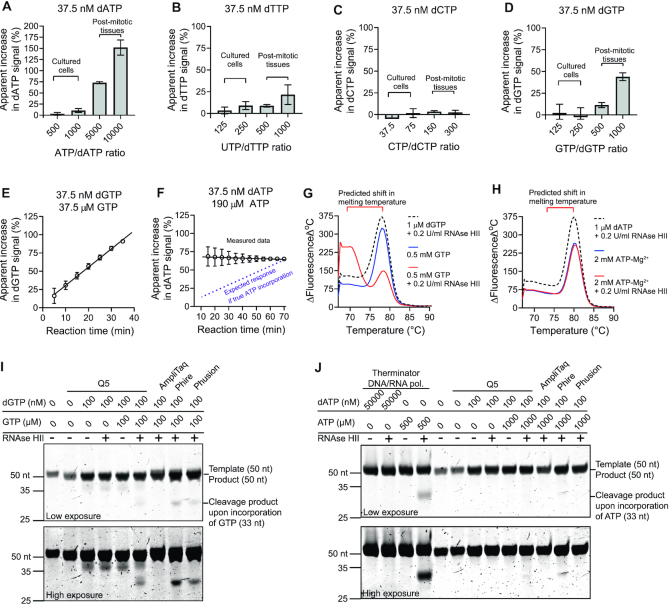
Assessment of interference by rNTPs. (**A–D**) The assay reactions (197-nt templates) were spiked with the indicated amounts of rNTPs and the change in the dNTP signal was measured. The concentrations shown are final reaction concentrations. The estimated upper ranges for rNTP/dNTP ratios are shown for cultured cell and post-mitotic tissues. (E, F) The effect of reaction time on the apparent interfering GTP (**E**) and ATP (**F**) signal. The purple dashed line represents the expected increase in the interfering signal assuming true ATP incorporation, essentially steady-state supply of ATP, and a time-dependent exhaustion of dATP during the reaction. Reaction time-independent increase in signal suggests trace amounts (∼0.015%) of dATP in the ATP preparation. (G, H) Melt curve analysis of RNAse HII-nicked 50-nt products of rGTP/dGTP (**G**) and rATP/dATP assay reactions (**H**). For reactions containing 2 mM ATP, 1.6 mM MgCl_2_ was added to compensate the chelation of Mg^2+^ by ATP. (I, J) Denaturing polyacrylamide (13%) gel electrophoresis of the reaction products with and without thermostable RNAse HII. The rNTP-incorporating *Thermococcus 9°N-7* mutant DNA polymerase (Therminator) was used as positive control for the ATP incorporation. Supplementary Figure S6 shows similar assessment of ATP incorporation using alkaline cleavage of ribonucleotide bonds. The error bars in the figures represent standard deviation and mean from three technical replicates.

Cellular rNTP concentrations are several dozen- to thousand-fold higher than the corresponding dNTPs, and most DNA polymerases can misincorporate rNTPs to some degree (13). Therefore, we estimated rNTP-to-dNTP ratios that could occur in cultured cells and post-mitotic tissues (5,14,15). Then, we tested how well our assay discriminates against purified commercially available rNTPs at such ratios (Figure 7). Adding purified rNTPs to the reactions to achieve typical rNTP-to-dNTP ratios present in cultured cells did not cause marked interference in the dNTP signals (Figure 7A–D). However, the much higher rNTP-to-dNTP ratios putatively present in post-mitotic tissue samples caused a clear increase in dATP and dGTP signals. Because commercial molecular biology-grade rNTP preparations derive from biological sources (generally of 98–99% purity), and may therefore contain trace amounts (∼0.015%) of dNTPs, it was essential to verify whether the observed rNTP interference was real or due to a small amount of contaminating dNTP in the rNTP preparations (added in huge excess to achieve the biologically relevant ratios). The constant excess of the rNTP, but gradual exhaustion of the corresponding dNTP, during the reaction should lead to a time-dependent increase in the interfering signal. This was indeed the case when we assessed dGTP in the presence of 1000-fold excess GTP, implying true interference (Figure 7E). However, this did not occur when we assessed dATP in the presence of 5000-fold excess ATP, suggesting that the interfering signal was not due to ATP incorporation (Figure 7F). To verify this, we utilized the 50-nt templates and performed the reactions in the presence of thermostable RNAse HII and subjected the products to melt curve and denaturing gel electrophoresis analyses. When the product strand contains a ribonucleotide opposite to the detections site, RNAse HII induces a nick that will cause lowering of the product melting temperature. Indeed, RNAse HII caused a clear shift in DNA melting temperature in the case of dGTP-detection reactions containing 0.5 mM GTP (Figure 7G). This shift did not occur in the dATP-detection reactions containing even 2 mM ATP (Figure 7H), indicating that ATP was not significantly incorporated. As a more traditional means of assessing rNTP incorporation, we also analysed the reaction products using denaturing gel electrophoresis. In this analysis, the cleavage of the ribonucleotide bond opposite to the detection site by RNAse HII is detectable as a 33-nt band when the 50-nt assay templates are used. The ribonucleotide bond-indicating cleavage product was clearly visible in the reactions containing 1000-fold excess GTP with Q5 and two other processivity-enhanced DNA polymerases (Phire and Phusion). Interestingly, a Taq polymerase seemed to incorporate GTP at a lower rate. In contrast, ATP incorporation by Q5 and the three other DNA polymerases were beyond the detection limit of 0.1 ng ssDNA (∼12 fmol of 33-nt cleavage product, Supplementary Figure S5) (Figure 7J). As a positive control for rNTP incorporation, a Thermococcus *9°N-7* mutant (D141A/E143A/A485L) DNA-RNA polymerase (available commercially as Therminator polymerase from New England Biolabs) readily incorporated ATP and gave a prominent cleavage product, which confirmed that the RNAse HII did cut this particular ribonucleotide bond. We also repeated the experiment with alkaline cleavage of ribonucleotide bonds with similar results (Supplementary Figure S6). In summary, only GTP caused a significant interference in the corresponding dNTP signal with Q5 polymerase.

### Elimination of rNTP interference by RNAse HII and ribonucleotide bond-specific shift in DNA melting temperature

**Figure 8.**
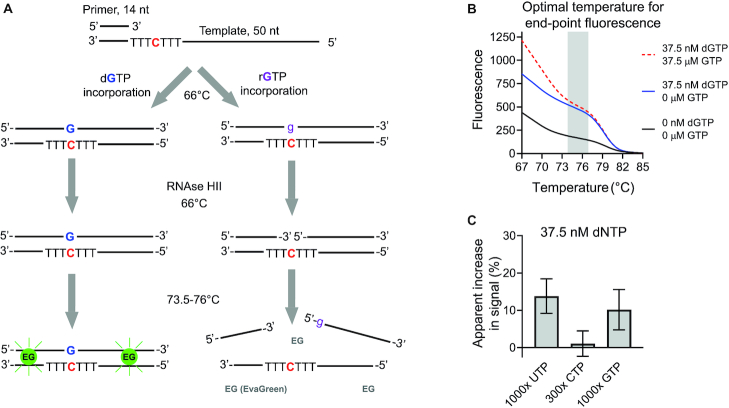
Utilization of thermostable RNAse HII and differential DNA melting temperature to eliminate interfering rNTP signal. (**A**) Schematic presentation of an assay modification to remove interfering rNTP signal. (**B**) Temperature-dependent decrease in interfering GTP signal in reactions containing 0.2 U/ml thermostable RNAse HII. Shaded grey area illustrates the optimal temperature range for the recoding of end-point fluorescence. (**C**) Apparent increase in dNTP signal in the presence of excess rNTP when the rNTP elimination modification was used. The bar graphs represent mean and standard deviation from three replicates.

We realized that the high specificity of EvaGreen for dsDNA allows the elimination of interfering rNTP signal by means of nicking the ribonucleotide bonds with a thermostable RNAse HII followed by reading the end-point fluorescence at a temperature at which the nicked DNA is denatured but the intact products remain double stranded (Figure 8A). We tested this approach with the 50-nt templates with which the cleavage of the ribonucleotide bond theoretically causes a 5–9°C decrease in the DNA melting temperature. The inclusion of thermostable RNAse HII in the reaction mixture and reading the end-point fluorescence between 73.5 and 76°C essentially abolished the interfering GTP signal (Figure 8B). We also utilized the same approach to reassess the increase in dNTP signal in the presence of excess UTP and CTP, two rNTPs with which the apparent interference was much slighter in comparison to GTP (Figure 8C).

### Optimization of sample preparation

As the starting point, we chose the widely used 60% methanol extraction (5–7,16,17). We also adopted a brief heat denaturation (5,7,16) and a 3-kDa cut-off centrifugal filtration (7) from previous studies to remove residual enzymatic activity-related interference (18) and any remaining macromolecules that could increase baseline fluorescence, and produce unspecific DNA polymerisation or polymerase inhibition. For enzymatic assays, the methanol extracts have typically been evaporated to dryness in vacuum. To minimize nucleotide hydrolysis, oxidation and deamination reactions, we chose a more rapid approach and extracted the methanol with diethyl ether. Similar diethyl ether washing steps have been validated for the extraction of several different nucleotides (19). The residual diethyl ether was easily removed by boiling the sample tubes cap open for 1 min or by a short evaporation (∼15 min) in a vacuum centrifugal evaporator at 65°C. The diethyl ether extraction step presumably also removes some putative non-polar DNA polymerase inhibitors such as hemin, bilirubin and bile acids based on the solubility of these compounds.

### Determination of dNTP concentrations in cultured cells

As a biological validation of the novel method, we measured dNTP levels from a mouse hepatoma cell line (Hepa1–6) cultured with or without 5 μM 5-fluorouracil, a thymidylate synthase inhibitor that blocks dTTP biosynthesis (Table 1). After a 6-hour treatment, 5-fluorouracil had depleted dTTP levels to one fifth of the levels in untreated cells. 5-fluorouracil also decreased dGTP and dCTP concentration to some extent, while that of dATP was increased. Several other cancer cells lines have shown similar pattern of changes in response to 5-fluorouracil (7,20). We also tested the effect of inhibition of pyrimidine biosynthesis by blocking mitochondrial respiratory complex III with 200 nM myxothiazol for 6 h. Complex III is required for the activity of dihydroorotate dehydrogenase (DHODH), a mitochondrial inner-membrane quinone oxidoreductase needed for the *de novo* biosynthesis of pyrimidine nucleotides (21). Myxothiazol decreased the levels of all dNTPs (Table 1), suggesting it halted cell proliferation as previously shown for lymphoblastic Jurkat T-cell line (22). This was dependent on complex III because the myxothiazol-induced decline in dNTPs was prevented by the expression of Pacific oyster alternative oxidase (PoAOX), an enzyme that can bypass the blockade of the complex III-IV segment of the respiratory electron transfer (Supplementary Figure S1B), as also previously shown by its heterologous expression in yeast (9).

**Table 1.** dNTP concentrations in cultured cells, pmol/10^6^ cells

Cell line	Treatment/condition	Transgene	dTTP	dATP	dCTP	dGTP
Mouse hepatoma (Hepa 1–6)	Untreated		42 ± 6.9	15 ± 2.3	133 ± 25	4.7 ± 0.8
	5 μM 5-fluorouracil		7.8 ± 0.9	24 ± 0.9	67 ± 7.2	1.4 ± 0.1
	Vehicle (0.008% EtOH)	empty vector	40 ± 2.5	18 ± 2.4	161 ± 13	7.5 ± 0.5
	200 nM myxothiazol	empty vector	3.8 ± 0.6	4.6 ± 1.5	3.5 ± 0.5	0.5 ± 0.4
	200 nM myxothiazol	*PoAOX*	43 ± 4.4	23 ± 3.8	163 ± 30	13 ± 3.8
Human colorectal carcinoma (HCT116)	n/a		16.7 ± 2.4	8.2 ± 0.9	5.3 ± 0.8	2.8 ± 0.5
Human neonatal skin fibroblasts	n/a		23 ± 2.8	10.8 ± 0.9	12.3 ± 0.8	4.9 ± 0.1
Mouse embryonic skin fibroblasts (NIH/3T3)	n/a		31.4 ± 2.1	8.1 ± 0.9	15 ± 1.6	4.7 ± 0.5

The values represent mean ± SD from three distinct cell culture flasks. The treatment time for 5-fluorouracil and myxothiazol was 6 h. The extract dilutions are shown in Supplementary Table S3. The 197-nt templates were used for these measurements.

### Determination of dNTP concentrations in mouse tissues

To demonstrate the true utility of our method, we measured dNTP concentrations in mouse liver, heart and skeletal muscle, tissues comprising mostly non-dividing cells and therefore having minimal dNTP concentrations. Our method was easily able to quantify all four dNTPs from these three tissues either with the 197-nt (Supplementary Table S4) or 50-nt templates (Table 2). With the 50-nt templates, we utilized the RNAse HII-based modification to remove putative rNTP-interference. The results were largely similar with and without this modification, except for dGTP in the heart, for which the rNTP-interference removal modification resulted in lower values (–46%, –4.5 SDs). We assessed the biological sample-related DNA polymerase inhibition by spiking the reactions with 0.25 pmol dNTPs and found that the detection efficiencies ranged from 90 to 100% (Supplementary Table S5). We also tested different extract dilutions and found only minimal inhibition (Supplementary Figure S7). Supplementary Figures S8–S10 show representative fluorescence values during the reaction phase, at endpoint, and also the melt curve analyses of the reaction products.

**Table 2.** dNTP concentrations in mouse tissues, pmol/mg tissue

	dTTP	dATP	dCTP	dGTP	Extract dilution [volume per initial tissue weight]
Liver	0.87 ± 0.22	0.20 ± 0.03	0.39 ± 0.08	0.51 ± 0.08	6 μl/mg
Heart	0.64 ± 0.17	0.20 ± 0.01	0.24 ± 0.04	2.3 ± 0.25	6 μl/mg: dTTP, dATP, dCTP 20 μl/mg: dGTP
Skeletal muscle	0.58 ± 0.13	0.16 ± 0.02	0.11 ± 0.04	0.46 ± 0.1	5 μl/mg

The values represent mean ± SD from four independent biological replicates. The dNTP concentrations were measured using the 50-nt templates and the rNTP-interference elimination step. The extract dilutions refers to the dilution before the assay.

Across the four tissues, dATP was the least abundant dNTP (∼0.2 pmol/mg tissue) while dTTP and dGTP concentrations were at least 2-fold higher (Table 2). Heart tissue showed the most asymmetric dNTP pools with dGTP concentration approximately 2 pmol/mg while the concentrations of other dNTPs were more similar to that in the liver and skeletal muscle.

### dNTP pool asymmetry in different samples and comparison to previously reported values

To obtain data comparable to previous studies (5,7,15), we measured dNTP concentrations in cultured human carcinoma cells (HCT116), mouse fibroblasts (NIH/3T3) and in primary human fibroblasts (Table 1). We compared the published data to our results (Table 3). In cultured mammalian cells, dTTP has been reported to comprise nearly 50% of the total dNTP pool, while dGTP has usually been the least abundant dNTP. We also found this pattern in the colorectal carcinoma cell line HCT116, and in cultured mouse and human fibroblasts. The mouse hepatoma cell line (Hepa1–6) showed a somewhat different pattern, with the dCTP comprising most of the dNTPs.

**Table 3.** dNTP pool sizes in various samples and comparison to previously published values

Sample	dTTP % (pmol/10^6^ cells or pmol/mg)	dATP % (pmol/10^6^ cells or pmol/mg)	dCTP % (pmol/10^6^ cells or pmol/mg)	dGTP % (pmol/10^6^ cells or pmol/mg)	Reference	Method
Mouse hepatoma cell line (Hepa 1–6)	22% (42)	7.5% (15)	68% (133)	2.4% (4.7)	This study	
Human neonatal skin fibroblasts	45% (23)	21% (11)	24% (12)	10% (4.9)	This study	
Human skin fibroblasts	47% (92)	22% (47)	21% (44)	9% (18)	Ferraro et al. 2010 (5)	Enzymatic and HPLC
Mouse skin fibroblasts (NIH/3T3)	53% (31)	14% (8.1)	25% (15)	8% (4.7)	This study	
Mouse skin fibroblasts (BALB/3T3)	41% (92)	20% (44)	31% (70)	8% (18)	Kong *et al.* 2018 (15)	HPLC-MS
Human colorectal carcinoma cell line (HCT116)	51% (17)	25% (8.2)	16% (5.3)	8% (2.8)	This study	
	41% (20)	28% (14)	21% (11)	10% (4.7)	Wilson *et al.* 2011 (7)	Enzymatic
Mouse liver	44% (0.87)	10% (0.2)	20% (0.39)	26% (0.51)	This study	
Mouse heart	19% (0.64)	6% (0.2)	7% (0.24)	68% (2.3)	This study	
	14% (0.2)	2% (0.02)	9% (0.13)	75% (1.0)	Olafsson *et al.* 2017 (6)	HPLC-MS
	21%	5%	7%	68%	Tran *et al.* 2019 (23)	HPLC-MS
Mouse skeletal muscle	44% (0.58)	12% (0.16)	8% (0.11)	35% (0.46)	This study	
	34% (0.34)	18% (0.18)	23% (0.23)	26% (0.26)	Olafsson *et al.* 2017 (6)	HPLC-MS
	15% (0.15)	33% (0.25)	20% (0.15)	32% (0.23)	Ylikallio *et al.* 2010 (31)	Enzymatic

The %-values represent individual dNTP levels normalized to total dNTP concentration. Absolute values (where applicable) are given in the brackets as pmol/10^6^ cells for cultured cells and as pmol/mg tissue for tissues.

We obtained HPLC-MS-quantified reference data for mouse heart and skeletal muscle from the studies by Olafsson *et al.* (6) and Tran *et al.* (23). Despite deviation in the absolute values, this reference data and our data showed highly similar dNTP pool asymmetry patterns for the mouse heart (Table 3). In this tissue, dGTP comprised ∼70% of the dNTP pool. In the mouse skeletal muscle, the dNTP pool was more symmetric according to our results and the published data.

In summary, apart from the inevitable variation that likely arises from the slightly different cell culture conditions, cell batches, extraction procedures and mouse strains in different laboratories, we found an excellent agreement between our and published results on dNTP levels across different sample types.

## DISCUSSION

Balanced dNTP pools are vital for efficient error-free DNA synthesis (1). The current knowledge related to regulation and fluctuations in dNTP pools derive mainly from cell culture experiments. Tissue dNTP pools in health and disease have been studied surprisingly little. This has likely been partly because of the low concentrations of dNTPs in tissue samples and the lack of a sensitive-enough methodology. To fill this methodological gap, we developed a simple robust method able to quantify each of the four canonical dNTP from mouse tissue biopsies.

Our novel and the published enzymatic fluorometric assay (7) share a number of benefits over the traditional methods based on radionucleotide incorporation. First, the quantification reaction takes place in a single microplate well and requires only pipetting of a reagent mix and the sample. Secondly, a typical qPCR instrument can be used for the temperature control and to read the fluorescence. Whereas in the case of radionucleotide-based detection, the reaction products have to be transferred onto filter paper, washed multiple times, and quantified using a scintillator. Such manual labour-requiring steps are prone to human error and limit the feasible number of samples that can be analysed simultaneously. In addition, handling of radioactive reagents requires permits and stringent safety measures. Recently, an improved enzymatic radionucleotide-based assay for dNTPs was developed in which biotinylated oligonucleotide templates are incorporated onto streptavidin-coated microplates to facilitate washing steps and recovery of the synthetized radio-labelled DNA (17). The inventors of this assay measured dNTPs from several cell lines, but they did not report suitability of the assay for tissue samples.

A novel innovation of our method is the utilization of the highly sensitive EvaGreen detection chemistry for dsDNA, and recent advances in chemical oligonucleotide synthesis that allow production of ultralong (∼200 nt) ssDNA templates at reasonable cost. EvaGreen is a well-characterized intercalating DNA fluorochrome that shows higher specificity for double-stranded versus ssDNA than the more traditional SYBR Green fluorochrome used in qPCR (12). Moreover, it can be used at saturating concentration without DNA polymerase inhibition. The fluorescence of EvaGreen is proportional to the molar concentration and length of dsDNA, and therefore the template length determines the signal amplification in our method. We found the maximum sensitivity with the ultralong 197-nt templates but even with the 50-nt templates the assay has sufficient sensitivity for most sample types. One benefit of EvaGreen-based detection as compared to fluorescent probes is that the choice of DNA polymerase is not limited to enzymes possessing 5′ to 3′ exonuclease activity to hydrolyse the probe. An appropriate DNA polymerase is crucial for the accurate quantification of dNTPs by the enzymatic method. For instance, the commonly used Klenow fragment can substitute the ribonucleosides GTP and CTP for the corresponding dNTPs to be quantified (5,24,25). We found that the Q5 DNA polymerase, which we mainly used in this work, retains specificity in the presence of rNTP-to-dNTP ratios typically present in cultured cells. However, we found this DNA polymerase capable of incorporating GTP at rates that may bias measurement to some degree from post-mitotic tissue samples. To overcome this, we took advantage of EvaGreen's high specificity for dsDNA and employed a thermostable RNAse HII to induce a shift in DNA melting temperature in the products containing a ribonucleotide bond. Due to the template design and given that the non-limiting dNTPs are provided in large excess, the interference from rNTPs is essentially limited to a single misincorporation at the detection site. Therefore, after the cleavage of the ribonucleotide bond by RNAse HII, the specific signal can be separated by reading the fluorescence at a temperature at which the specific full-length products remain dsDNA but the unspecific products denature to ssDNA. For even more stringent specificity, it is also possible to perform melt curve analysis and use the rate of fluorescence change at the specific temperature as a readout. A downside of the rNTP interference removal protocol is that the maximum template length is limited to approximately 70 nt which decreases the sensitivity to some degree. However, even with 50-nt templates we found more than sufficient sensitivity to assess dNTPs in tissue extracts. In addition to the GTP interference, we found an apparent increase in dATP signal in the presence of 5000–10 000-fold excess ATP. Measuring dATP in the presence of such high excess of ATP is, however, problematic as even as low as 0.015% contaminating dATP in the ATP preparations would explain the apparent increase in signal. Therefore, we investigated this apparent interference by several means, but we did not find any signs of significant ATP incorporation by the tested DNA polymerases.

In addition to the discrimination against rNTPs, the DNA polymerase of choice has to be able to tolerate a considerable amount of inhibitory impurities, especially in the case of concentrated tissue extracts. To our knowledge, the utility of modern inhibitor-resistant high-fidelity DNA polymerases in the enzymatic determination of dNTPs has not been investigated before, even though the enzyme inhibition is a potential source of error and the DNA polymerase fidelity may affect considerably the background signal. We found that the previous (7) fluorometric assay based on probes and AmpliTaq DNA polymerase was highly sensitive to inhibition by concentrated tissue and cell extracts that posed no problem for the processivity-enhanced Q5 DNA polymerase and the EvaGreen detection chemistry. During the revision of this manuscript Szabo *et al.* reported similar problems with the original fluorometric assay and found unspecific probe hydrolysis as the source of unstable background (26). Moreover, they identified differential inhibition of exonuclease and polymerase activity of Taq DNA polymerases by biological samples as the major source of the interference. They proposed a mathematical correction for the problem, but we did not have the opportunity test it for this study.

The highly unbalanced dNTPs required by the enzymatic methods may lead to eventual incorporation of wrong deoxyribonucleotides with a concomitant elevation of background signal. We, indeed, found that the DNA polymerase with the highest rated fidelity gave the lowest background. In fact, in the absence of the dNTP to be quantified no full-length products were visible in melt curve analyses of dATP and dTTP detection reactions with the Q5 DNA polymerase. In the case of dGTP detection reactions the background-related full-length products were detectable suggesting a slightly higher error-rate by the enzyme or traces of dGTP in the detection reagents. However, the polymerase error-rate was clearly not the sole factor determining the background. In fact, we found that the EvaGreen-based quantification robustly worked with four different DNA polymerases without any prior optimization. The assay was also robust with respect to modest alterations in the reaction conditions such as temperature, template concentration and length, and enzyme amount. Importantly, after optimization, we verified reliable quantification down to 30 fmol using 197-nt templates and 90 fmol using 50-nt templates. In fact, the true LLOQ values are likely to be lower than these are, especially, if the product melting temperature profile is employed for the calculation of the end-point readout. As a comparison, a radionucleotide-based method has been reported to reach a 100 fmol quantification limit (data not shown and criteria for LLOQ not reported) (5). The most sensitive published HPLC-MS system has a LLOQ of 60 fmol, and typically the LLOQ values for chromatographic methods range from 0.25 to 10 pmol (6). The previous fluorometric assay based on double-quenched probes had LLOQ values ranging from 0.8 to 1.3 pmol (7). Later, the same authors published an addendum describing a triple-quencher approach that decreases the LLOQ to 0.1 pmol for dTTP. Less stringent criteria (signal-to-noise ratio of 5) for LLOQ was, however, used by these authors than by us. Recent findings have also questioned the robustness of the probe-based detection with biological samples (26). Recently, a rather elaborate enzymatic two-stage dNTP detection involving a fluorescent probe, two DNA fragments, two different DNA polymerases and a DNA ligase was developed (27). This assay could reach a detection limit that is orders of magnitude lower than any other existing method could. However, this complex assay was developed for single-molecule DNA sequencing and its utility remain undemonstrated with biological samples.

The main reason we set out to develop a new method was the need to quantify minute dNTP concentrations from reasonably small mouse tissue biopsies. The first enzymatic assay for dNTPs was published 1969 (28). Since then, small improvements to increase sensitivity and specificity have been reported (4,5,17,29). All these studies have, however, included only cultured cells as a biological sample. Yet, enzymatic methods have been used to measure dNTP concentrations from liver (30) and skeletal muscle samples (31–33), but no method validation data for tissue samples have been reported in these studies. In skeletal muscle samples, individual dNTP concentrations have ranged from less than 0.02 to 0.3 pmol/mg with the biggest variation seen in dATP levels (31,32). Interestingly, a HPLC-MS-based method has also shown surprisingly low dATP concentrations for mouse heart tissue (0.02 pmol/mg) (6). Although the ribonucleotide ATP is the physiological substrate for cellular ATPases, several of these enzymes can use dATP instead of ATP (34,35). We speculate that the variation in dATP levels could be related to ischemia during sample collection and delayed quenching of enzymatic activity. Similar to two chromatographic quantifications (6,23), we found heart dGTP concentration to be several-fold higher than that of other three dNTPs. Mitochondrial dNTP pools are extremely asymmetric with dGTP concentration being more than 10-fold higher than the concentration of other dNTPs (16). This asymmetry was reported to be especially notable in heart mitochondria (155-fold excess dGTP) (16). Therefore, it is obvious that in mitochondria-rich tissues such as in heart, dGTP would be the most abundant dNTP. In summary, according to our results and the published literature the tissue dNTP concentrations are generally low; only a fraction of a picomole per mg of tissue.

We acknowledge the following points as room for further development of the assay. We chose the methanol-based extraction because of its wide use in previous studies (5–7,16,17). However, to our knowledge, no systematic comparison of different extraction procedures for dNTPs exists in the literature. We optimized the key parameters of the dNTP assay for Q5 DNA polymerase. However, this particular DNA polymerase is not a requirement for the assay and other DNA polymerases may also be used with minimal adjustments. We mainly optimized the assay for maximal sensitivity at low inputs. If necessary, the dynamic range could be adjusted by varying the number of detection sites in the template, as done for the probe-based assay (7), or using a higher template concentration. Interference from dUTP is an unlikely source of error for most sample types because of the high cellular dTTP-to-dUTP ratios (36–40). However, in the case of potential dUTP accumulation e.g. due to the inhibition of dUTP catabolism or thymidylate synthesis, the extracts can be digested with dUTPase to remove the potential interference (7,40,41). In theory, it is also possible to quantify dUTP by measuring extracts with and without UTPase digestion and using a readily dUTP-incorporating DNA polymerase. As with all enzymatic dNTP assays, enzyme inhibition and rNTP misincorporation are potential sources of error. Extended reaction times may mitigate the enzyme inhibition but increase the probability of rNTP incorporation. If necessary, the simple modification to eliminate rNTP interference, as presented here, should be employed. The assay produces a non-linear standard curve at high input. Thus, if inhibition is present, the Standard Addition Method is valid only after a careful titration of appropriate sample input. Some samples may show intrinsic fluorescence. Therefore, we recommend subtraction of baseline fluorescence from the end-point fluorescence values. This correction also decreased well-to-well variation. An approximate cost of our assay, excluding sample preparation, is 34 EUR (37 USD) per 100 detection reactions with 197-nt templates.

In summary, we developed an enzymatic fluorometric assay that is simple, inexpensive, and most importantly has the sensitivity and robustness to measure dNTPs from non-proliferating cells or from tissue extracts. Without technical replicates, less than 4 mg tissue or 0.1 × 10^6^ cultured cells is sufficient. Moreover, 96 and 384-well formats of the assay allow a large number of samples to be measured simultaneously. Only a handful of studies have addressed the topic of tissue dNTP levels in health and disease (6,30–32). As nucleotide metabolism is increasingly studied in the field of cancer biology and in relation to metabolic diseases such as mitochondrial disorders (32,42) the availability of an easily applicable dNTP assay for use in the general laboratory is of the utmost importance.

## Supplementary Material

gkaa516_Supplemental_Files
