# Supplementary material for: A sensitive assay for dNTPs based on long synthetic oligonucleotides, EvaGreen dye and inhibitor-resistant high-fidelity DNA polymerase
Source: Nucleic Acids Res. 2020 Jun 23;48(15):e87. doi: 10.1093/nar/gkaa516 (PMC7470940; doi:10.1093/nar/gkaa516)
Supplement: gkaa516_Supplemental_Files [file gkaa516_supplemental_files.zip › Step-by-step protocol.pdf]

Step-by-step protocol for measurement of dNTP concentrations in mouse tissues

Reagents and materials

- Ice-cold 60% MeOH

- Amicon Ultra-0.5-ml centrifugal filters, 3kDa (Merck, UFC500396). Mark and weight the collection tubes before use. Equilibrate with 60% MeOH before use! Do not let dry!

- Diethyl ether. Intoxicating, flammable and explosive! Open diethyl ether containing bottles and vials in fume hood! Check the expiry date! Do not use expired diethyl ether.

- DNA oligomers (Supplementary Table 1)

Dissolve templates to concentration of 10 µM (e.g. 2.5 nmol DNA and 250 µl H_2O). Dissolve primer to concentration of 100 µM, and from this prepare a working solution of 10 µM. Store DNA oligos in aliquots at -20ºC.

- NEB Q5 Hot-Start High-Fidelity DNA polymerase

- dNTP Set 100 mM Solutions (e.g. Thermo R0181)

- 2.5 mM dNTP mixes:
  dGTP + dCTP + dTTP mix for dATP detection:
  5 µl of 100 mM dGTP
  5 µl of 100 mM dCTP
  5 µl of 100 mM dTTP
  185 µl of H_2O

  dCTP + dTTP+ dATP mix for dGTP detection:
  5 µl of 100 mM dCTP
  5 µl of 100 mM dTTP
  5 µl of 100 mM dATP
  185 µl of H_2O

  dGTP + dTTP+ dATP+ mix for dCTP detection:
  5 µl of 100 mM dGTP
  5 µl of 100 mM dTTP
  5 µl of 100 mM dATP
  185 µl of H_2O

  dGTP + dCTP+ dATP+ mix for dTTP detection:
  5 µl of 100 mM dGTP
  5 µl of 100 mM dCTP
  5 µl of 100 mM dATP
  185 µl of H_2O

- dNTP standards: Eight-point 1:2 serial dilution covering 400 to 0 nM. This can be prepared from individual dNTP solutions or from a dNTP mix.

dNTP extraction from mouse tissues

Tissue samples should be snap-frozen as fast as possible after cervical dislocation or induction of the terminal anesthesia of the mice. The recommended amount of tissue is 20 mg for liver.

1. Homogenize tissue in 550 µl of ice-cold 60% MeOH (up to 40 mg of tissue).

   Liver and other soft tissues:
   First completely crush the tissue using a microtube pestle. Then use a battery-operated microtube pestle homogenizer for 30s.

   Cut fibrous tissues such as heart and skeletal muscle into small pieces on dry ice. Transfer the tissue pieces into a roughened glass-to-glass potter tissue grinder. Homogenize using 20-30 rotating strokes or until the solution is fully homogenous [Note1].

2. Incubate 3 min at 95ºC. Cool down on ice.
3. Centrifuge 18500g for 6 min at +4ºC.
4. Collect supernatant (max 550 µl) into equilibrated Amicon Ultra-0.5-ml centrifugal filter. The collection tube has to be pre-weighted!
5. Centrifuge 15000g 60 min at +4ºC and save the flow-through. ~50 µl sample will remain in the column. The liquid level of remaining solution should be below the filter line. The volume of the remaining liquid in the column can be measured after spinning the column upside down in a new empty tube.
6. [Optional] Because the collection tubes of the Amicon centrifugal filters have relatively loose caps, we recommend transferring the filtered extract into new standard 2-ml Eppendorf tubes before the next step.
7. Add 1.4 ml ice-cold diethyl ether. Vortex 30s. [Note2]
8. Centrifuge 15000g 1s. Discard most of the upper layer.
9. Repeat step 7 and 8.
10. Concentrate the sample and evaporate residual diethyl ether using Speed-Vac: High setting (65ºC) for 15 min.
11. Determine the amount of remaining solution by weighing the tubes (1 mg = 1 µl). Adjust sample volume to 6 µl per mg initial tissue weight.
12. Store the extracts at -80ºC.

dNTP measurement procedure with 197-nt templates

Prepare a master mix for a dNTP to be quantified (dATP, dGTP, dCTP or dTTP). Equilibrate the master mix containing all components except the polymerase to room temperature. Add polymerase into the master mix immediately before use. Briefly vortex and spin down.

For dATP and dTTP
2x master mix recipe for 100 reactions with final volume of 10 µl

167.5 µl of H_2O
200 µl of 5X Q5 reaction buffer
27.5 µl of 10 µM nucleotide detection primer
25 µl of 10 µM template
20 µl of 2.5 mM dNTP mix without the dNTP to be quantified.
50 µl of 20X EvaGreen stock
10 µl of 2000 U/ ml Q5® High-Fidelity DNA Polymerase

Final concentrations:
Primer, 0.275 µM
Template, 0.25 µM
Non-limiting dNTPs, 50 µM
1 X EvaGreen
Q5 polymerase, 20 U/ml

For dGTP and dCTP
2x master mix recipe for 100 reactions with final volume of 10 µl

172.5 µl of H_2O
200 µl of 5X Q5 reaction buffer
27.5 µl of 10 µM nucleotide detection primer
25 µl of 10 µM template
20 µl of 2.5 mM dNTP mix without the dNTP to be quantified.
50 µl of 20X EvaGreen stock
5 µl of 2000 U/ ml Q5® High-Fidelity DNA Polymerase

Final concentrations:
Primer, 0.275 µM
Template, 0.25 µM
Non-limiting dNTPs, 50 µM
1 X EvaGreen
Q5 polymerase, 10 U/ml

Assay samples preferably in triplicates in a 384-well qPCR plate with white wells.

1. Place the qPCR plate on ice.
2. Pipet in reverse mode 5 µl of master mix into the wells.
3. Pipet 5 µl of sample or standards.
4. Program a qPCR instrument to perform the following steps:
   Step 1: 10s at 95ºC.
   Step 2: 1s 75ºC
   Step 3: read baseline fluorescence (FAM/SYBR Green channel of the instrument) [Note3].
   Step 4: 1s at 66ºC
   Step 5: read fluorescence. [Note4]
   Step 6: 5min at 66ºC
   Repeat step 4 to 6 for 10 cycles for dATP, 7 cycles for dTTP and dCTP, and 3 cycles for dGTP.
   Step 7: 5s 75ºC [Note5]
   Step 8: read end-point fluorescence.

5. Turn off the automatic baseline correction by the instrument. Export the raw fluorescence values for data analysis.
6. Generate a sigmoidal standard curve from the baseline-corrected end-point fluorescence values of the standard samples. Interpolate the concentrations of the samples.

[Note 1] Incomplete homogenization may lead to a severe underestimation of dNTP pools in muscle tissues.

[Note 2] Diethyl ether extracts methanol and hydrophobic metabolites (free heme, bilirubin etc.)

[Note 3] The first fluorescence measurement gives the baseline, which should be subtracted from end-point fluorescence.

[Note 4] The steps 4 to 6 allow monitoring of the fluorescence during the reaction phase.

[Note 5] The elevation of temperature to 75ºC dissociates the unused primers reducing the background fluorescence.

Modifications for 50-nt templates with rNTP interference discrimination

- 20 kU/ml Thermostable RNAse HII (IDT, 11-02-12-01). Dilute a 2 U/ml working solution in dilution buffer provided by the manufacturer. The working solution can be stored in aliquots at -20ºC. Note that the enzyme requires at least 0.01% non-ionic detergent for maximal activity (provided by the dilution buffer).

For dATP and dTTP
2x master mix recipe for 100 reactions with final volume of 10 µl

67.5 µl of H_2O
200 µl of 5X Q5 reaction buffer
27.5 µl of 10 µM nucleotide detection primer
25 µl of 10 µM template
20 µl of 2.5 mM dNTP mix without the dNTP to be quantified.
50 µl of 20X EvaGreen stock
100 µl of 2 U/ml RNAse HII
10 µl of 2000 U/ ml Q5® High-Fidelity DNA Polymerase

Final concentrations:
Primer, 0.275 µM
Template, 0.25 µM
Non-limiting dNTPs, 50 µM
1 X EvaGreen
0.2 U/ml RNAse HII
Q5 polymerase, 20 U/ml

For dGTP and dCTP
2x master mix recipe for 100 reactions with final volume of 10 µl

72.5 µl of H_2O
200 µl of 5X Q5 reaction buffer
27.5 µl of 10 µM nucleotide detection primer
25 µl of 10 µM template
20 µl of 2.5 mM dNTP mix without the dNTP to be quantified.
50 µl of 20X EvaGreen stock
100 µl of 2 U/ml RNAse HII
5 µl of 2000 U/ ml Q5® High-Fidelity DNA Polymerase

Final concentrations:
Primer, 0.275 µM
Template, 0.25 µM
Non-limiting dNTPs, 50 µM
1 X EvaGreen
0.2 U/ml RNAse HII
Q5 polymerase, 10 U/ml

- Program the qPCR instruments as instructed for 197-nt templates with following modifications:
  1) Change the temperature for baseline and end-point fluorescence to 75ºC for dATP and dCTP, 78ºC for dTTP, and 73.5ºC for dGTP
  2) Change the duration of the reaction phase to 50 min for dATP, 25 min for dCTP and dTTP, and 15 min for dGTP.
