# Supplementary material for: A sensitive assay for dNTPs based on long synthetic oligonucleotides, EvaGreen dye and inhibitor-resistant high-fidelity DNA polymerase
Source: Nucleic Acids Res. 2020 Jun 23;48(15):e87. doi: 10.1093/nar/gkaa516 (PMC7470940; doi:10.1093/nar/gkaa516)
Supplement: gkaa516_Supplemental_Files [file gkaa516_supplemental_files.zip › Supplementary Tables and Figures.pdf]

Supplementary Table 1. DNA oligonucleotides used in dNTP assays

	Sequences in 5' to 3' direction
**EvaGreen-based detection**	
Detection primer	CCGCCTCCACCGCC
dTTP detection template, 197nt	TCGCTCGCTCTTGCCTCGGTCCTCGCTCGCTCTTGCCTCGGTCCTCGCTCGCTCTTGCCTCGGTCCTCGCTCGCTCTTGCCTCGGTCCTCGCTCGCTCTTGCCTCGGTCCTCGCTCGCTCTTGCCTCGGTCCTCGCTCGCTCTTGCCTCGGTCCTCGCTCGCTCTTGCCTCGGTCCTTTATTT**GGCGGTGGAGGCGG**
dATP detection template, 197nt	AGACAGACACAAGACACAGACCAGACAGACACAAGACACAGACCAGACAGACACAAGACACAGACCAGACAGACACAAGACACAGACCAGACAGACACAAGACACAGACCAGACAGACACAAGACACAGACCAGACAGACACAAGACACAGACCAGAGAGACACAACAGACGGAGGAAATAAA**GGCGGTGGAGGCGG**
dCTP detection template, 197nt	CCACTCACTCTTACCTCAATCCCCACTCACTCTTACCTCAATCCCCACTCACTCTTACCTCAATCCCCACTCACTCTTACCTCAATCCCCACTCACTCTTACCTCAATCCCCACTCACTCTTACCTCAATCCCCACTCACTCTTACCTCAATCCCCACTCACTCTTACCTCAATCCTTTGTTT**GGCGGTGGAGGCGG**
dGTP detection template, 197nt	GGAGTGAGTGTGAGGTGAATGATGAGTGAGTGTGAGGTGAATGTAGAGTGAGTGTGAGGTGAATGATGAGTGAGTGTGAGGTGAATGTAGAGTGAGTGTGAGGTGAATGATGAGTGAGTGTGAGGTGAATGTAGAGTGAGTGTGAGGTGAATGATGAGTGAGTGTGAGGTGAATGGTTTCTTT**GGCGGTGGAGGCGG**
dTTP detection template, 50nt	TCGGTCCTCGCTCGCTCTTGCCTCGGTCCTTTATTT**GGCGGTGGAGGCGG**
dATP detection template, 50nt	ACAGACCAGAGAGACACAACAGACGGAGGAAATAAA**GGCGGTGGAGGCGG**
dCTP detection template, 50nt	TCAATCCCCACTCACTCTTACCTCAATCCTTTGTTT**GGCGGTGGAGGCGG**
dGTP detection template, 50nt	TGAATGATGAGTGAGTGTGAGGTGAATGGTTTCTTT**GGCGGTGGAGGCGG**
**Probe-based detection**	
Detection primer	CCGCCTCCACCGCC
dTTP probe	**FAM**/AGGACCGAG/**ZEN**/GCAAGAGCGAGCGA/**IBFQ**
dATP probe	**FAM**/TGGTCCGTG/**ZEN**/GCTTGTGCGTGCGT/**IBFQ**
dCTP probe	**FAM**/AGGATTGAG/**ZEN**/GTAAGAGTGAGTGG/**IBFQ**
dGTP probe	**FAM**/ACCATTCAC/**ZEN**/CTCACACTCACTCC/**IBFQ**
dTTP detection template	TCGCTCGCTCTTGCCTCGGTCCTTTATTT**GGCGGTGGAGGCGG**
dATP detection template	ACGCACGCACAAGCCACGGACCAAATAAA**GGCGGTGGAGGCGG**
dCTP detection template	CCACTCACTCTTACCTCAATCCTTTGTTT**GGCGGTGGAGGCGG**
dGTP detection template	GGAGTGAGTGTGAGGTGAATGGTTTCTTT**GGCGGTGGAGGCGG**

The pimer-binding sites are shown as bolded black letters at 3' end. Red letters shows the locations of dNTP-detection sites. The probe-binding sites are marked with blue letters.
FAM, 5(6)-carboxyfluorescein
ZEN, a proprietary quencher by Integrated DNA Technologies
IBFQ (Iowa Black FQ), a proprietary quencher by Integrated DNA Technologies

Supplementary Table 2. Critical reagents, materials and intrumentation

	Manufacturer	Catalogue number
**Reagents**		
MeOH (analytical grade)	Fisher Scientific	M/4000/PC17X
Stabilized diethyl ether (less than 1 year old)	Sigma	296082
100 mM dNTP solutions	Thermo Fisher	R0181
20x EvaGreen	Biotium	31000
Q5 High-Fidelity DNA polymerase*	New England Biolabs	M0491
Q5 Hot Start High-Fidelity DNA polymerase*	New England Biolabs	M0493
AmpliTaq Gold DNA polymerase	Thermo Fisher	N8080241
Phire Hot Start DNA II DNA polymerase	Thermo Fisher	F122
Phusion High-Fidelity DNA polymerase	Thermo Fisher	F530
Ribonucleotide Solutio Set	New England Biolabs	N0450
Thermostable RNAse H2	Integrated DNA Technologies	11-02-12-01
**Materials**		
Amicon Ultra-0.5 Centrifugal Filter Unit, 3 kDa	Merck	UFC500396
Hard-Shell® 384-Well PCR Plates, thin wall, skirted, black/white	Bio-Rad	HSP3865
Microseal 'B' PCR Plate Sealing Film	Bio-Rad	MSB1001
**Instrumentation**		
Speed-Vac Plus SC110A evaporator	Savant Instruments	
CFX384 qPCR instrument	Bio-Rad	

DNA oligonucleotides are listed in Supplementary Table 1.
* No difference in performance was observed between these two DNA polymerase preparations.

Supplementary table 3 (related to Table 1). Extract dilutions in dNTP measurements from cultured cells

Cell line	Treatment/condition	Transgene	Extract dilutions, µl/10^6 cells			
			dTTP	dATP	dCTP	dGTP
Mouse hepatoma (Hepa 1-6)	Untreated		100	100	400	100
	5 µM 5-fluorouracil		100	100	400	100
	Vehicle (0.008% EtOH)	empty vector	100	100	400	100
	200 nM myxothiazol	empty vector	100	100	100	100
	200 nM myxothiazol	*poAOX*	100	100	400	100
Human colorectal carcinoma (HCT116)	n/a		62	62	62	62
Human neonatal skin fibroblasts	n/a		190	190	190	190
Mouse embryonic skin fibroblasts (NIH/3T3)	n/a		120	120	120	120

The values given refer to preassay diutions. In the assay the extract volume was half of the reaction volume.

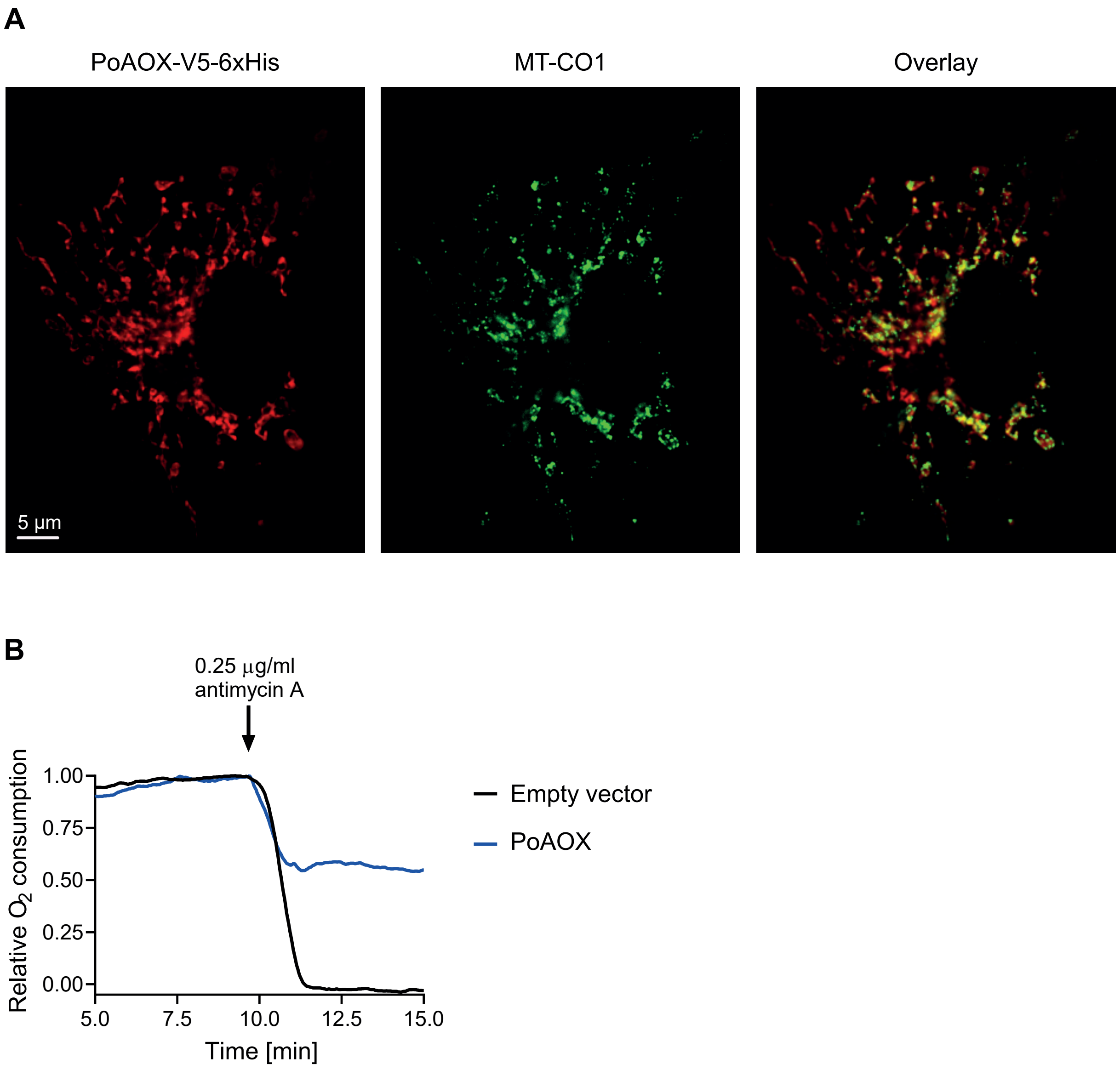

Supplementary Figure 1. Pacific oyster (*Crassostrea gigas*) alternative oxidase (PoAOX) localizes to mitochondria and confers antimycin A-resistant respiration in mammalian cells. (**A**) Transfected COS-1 cells immunostained for V5-tagged PoAOX and the endogenous mitochondrial protein MT-CO1 (cytochrome c oxidase subunit 1). (**B**) Respiration by intact Hepa1-6 cells in complete DMEM at +37°C was measured using Oxygraph-2k (OROBOROS instruments). Alternative oxidase activation was assessed by the addition of the respiratory complex III inhibitor antimycin A. Residual oxygen consumption after the subsequent addition of rotenone was set as background. Maximal basal respiration was set as another reference state and the data were scaled according to these two reference states.

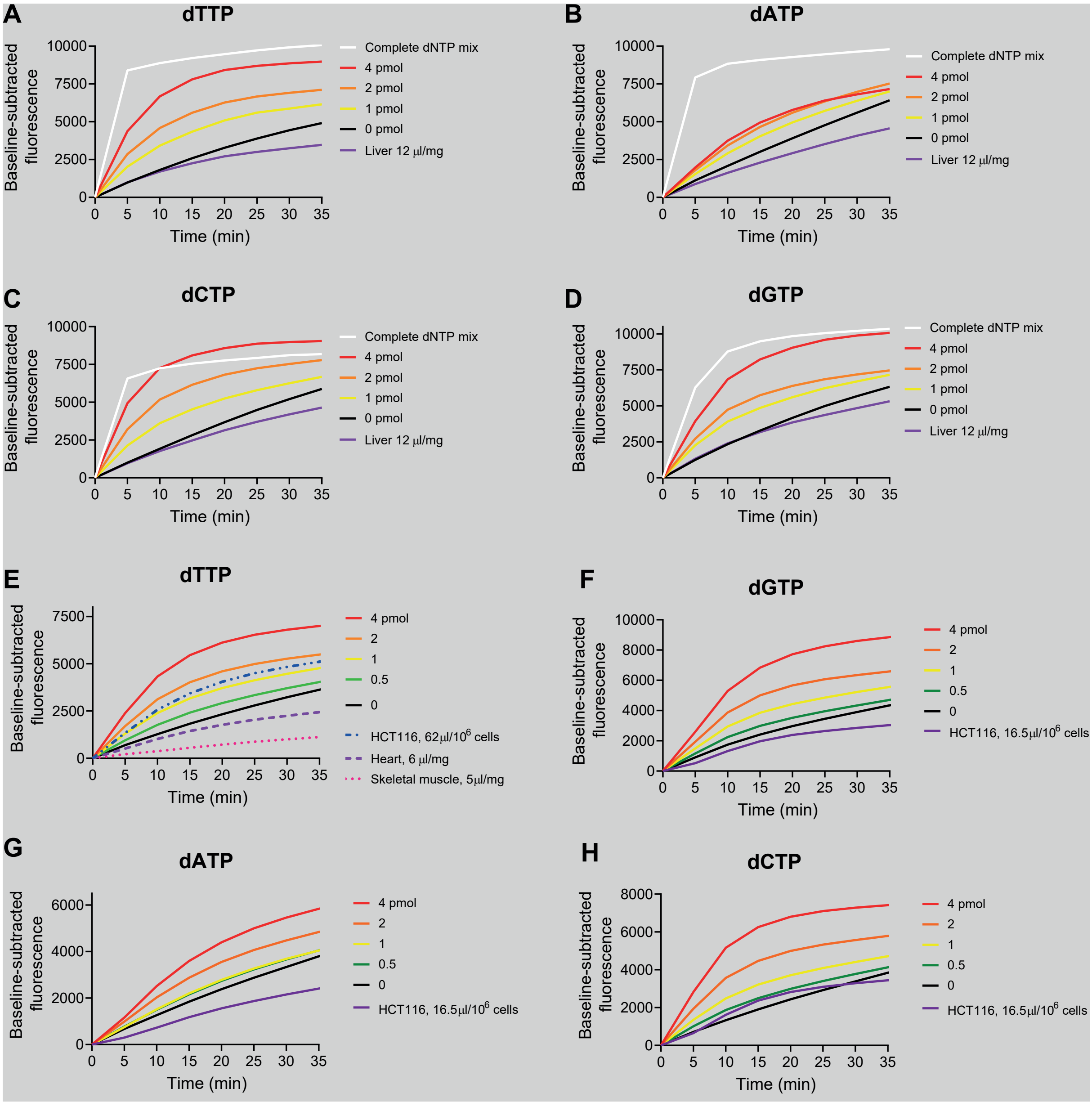

Supplementary Figure 2. Suitability of AmpliTaq Gold and probe hydrolysis-based dNTP quantification for tissue extracts. The protocol published by Wilson et al. was used to assess dNTPs from tissue and cell extracts. (**A-D**) Even relative dilute liver extracts (12 µl/mg of initial tissue weight) interfered with the assay leading to signal that was lower than the background (black curves, 0 pmol of purified dNTP, see Fig. 1C for comparison). (**E**) Heart and skeletal muscle extracts also interfered with the probe-based detection, whereas dTTP was measurable from dilute (62 µl/10^6 cells) HCT116 cell extracts. (**F-H**) Concentrated (16.5 µl/10^6 cells) HCT116 cell extracts interfered with the probe-based assay, leading to signal below background (black curves, 0 pmol of purified dNTP).

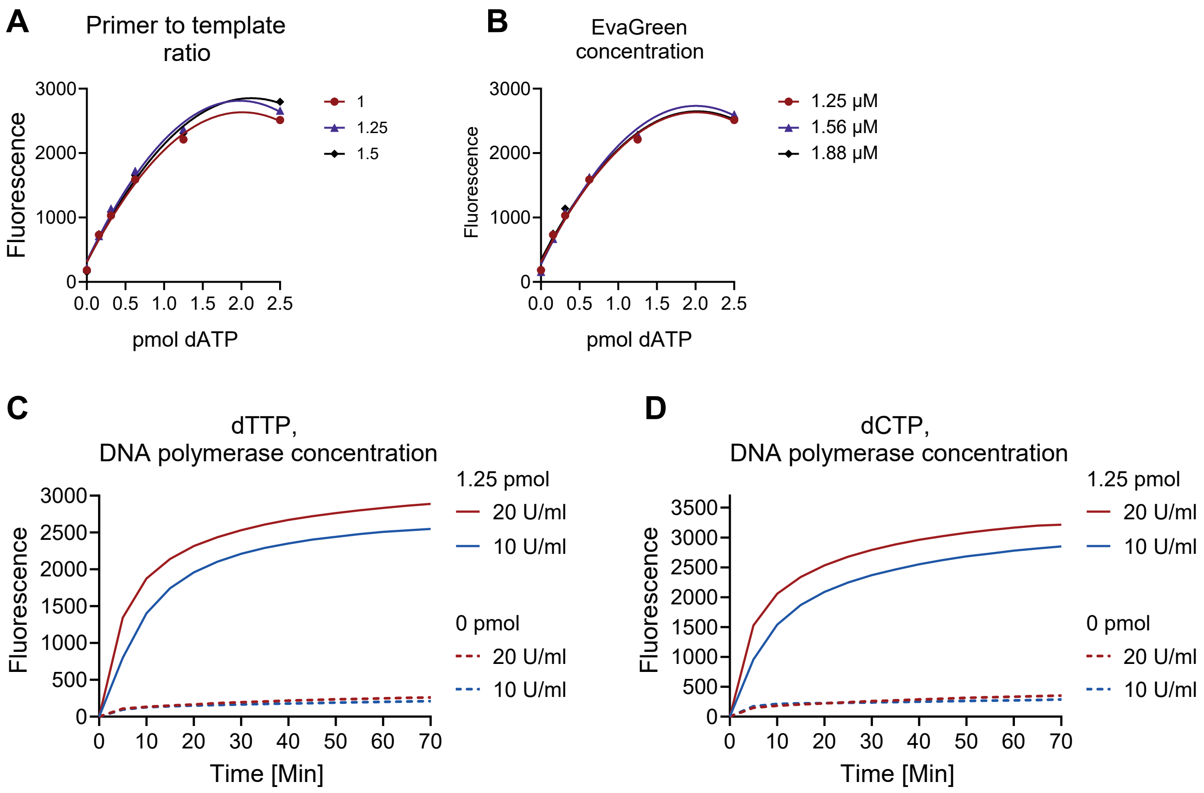

Supplementary Figure 3 (Related to figure 4). (**A**) Effect of primer-to-template ratio on assay performance at a reaction temperature of 68°C. (**B**) Increasing the EvaGreen concentration above the manufacturer's recommended concentration was of no benefit. (**C-D**) Effect of Q5 DNA polymerase concentration on dTTP (**B**) and dCTP (**C**) quantification.

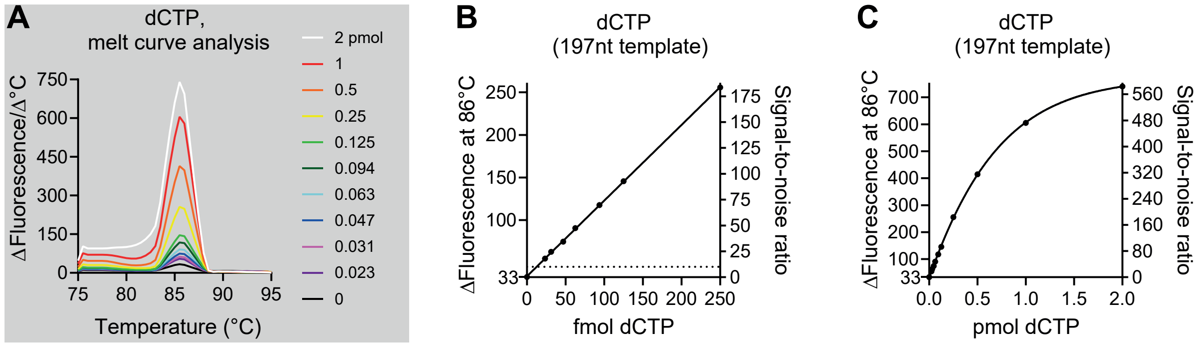

Supplementary Figure 4. Alternative generation of standard curves from melt curve analysis data. (**A**) Temperature-dependent change in fluorescence. (**B-C**) Standard curves generated from the rate of fluorescence change at 86°C. Linear regression was applied for the low range (**B**) and sigmoidal curve fit for the full range (**C**). The dashed line represents signal-to-noise ratio of 10.

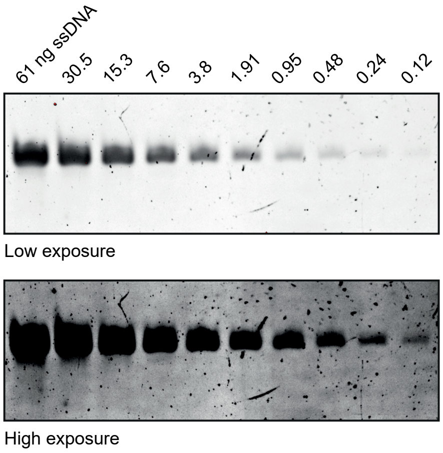

Supplementary Figure 5. Estimation of detection limit of GelRed staining for ssDNA in denaturing urea-PAGE. A serial dilution of a 50 nt ssDNA fragment (dCTP detection template) was stained after gel electrophoresis.

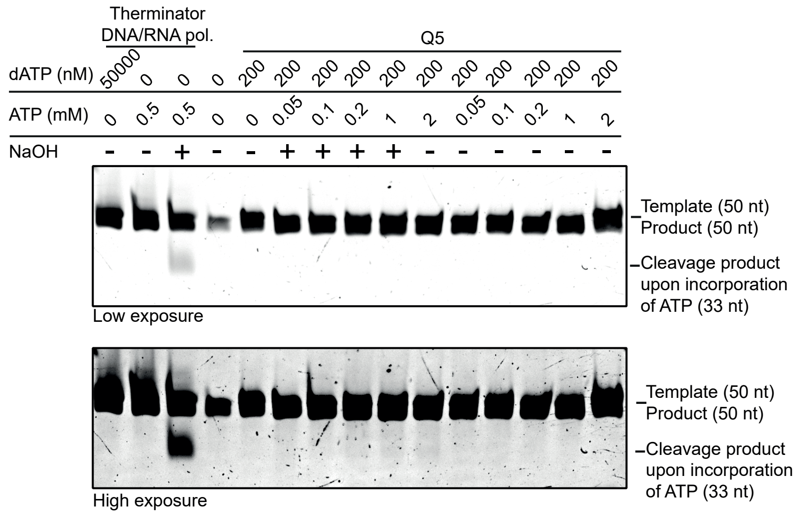

Supplementary Figure 6. Denaturing gel electrophoresis of reaction products after alkaline cleavage of ribonucleotide bonds. Reactions with a volume of 40 µl were run for 1h 15 min. To maintain approximately constant free Mg^{2+} concentration during the reactions, 0.8 mol $MgCl_2$ per 1 mol ATP were added. The reactions were stopped by addition of 6 mM EDTA. One 20-µl aliquot was incubated in 0.2 M NaOH at 55°C for 2 h, followed by neutralization with HCl. Another 20-µl aliquot was incubated in 0.2 M NaCl. After addition of equal volume of loading buffer (8M urea, 12% FICOLL and 0.01% bromophenol blue in TBE) and denaturation at 95°C the samples of 30 µl were resolved in pre-heated 15% polyacrylamide 8M Urea TBE gels. The Therminator DNA/RNA polymerase was used as a positive control for successful ribonucleotide bond cleavage.

Supplementary Table 4. Measurement of dNTP concentrations in mouse tissues using 197-nt templates, pmol/mg tissue

	dTTP	dATP	dCTP	dGTP	Extract dilution [volume per initial tissue weight]
Liver	0.54 ± 0.13	0.21 ± 0.02	0.39 ± 0.09	0.47 ± 0.06	4 µl/mg: dTTP, dCTP and dGTP<br>3 µl/mg: dATP
Heart	1.06 ± 0.10	0.21 ± 0.05*	0.37 ± 0.09	4.3 ± 0.44	4 µl/mg: dTTP, dATP*, dCTP<br>28 µl/mg: dGTP
Skeletal muscle	0.66 ± 0.08	0.17 ± 0.01*	0.17 ± 0.03	0.42 ± 0.04	4 µl/mg: dTTP, dATP*, dCTP and dGTP

Tha values represent mean ± SD from four independent biological replicates.
* Heart and skeletal muscle dATP concentrations were measured with 2 µl/mg and 4 µl/mg extract dilutions with less than 6.1% discrepancy between the measurement. The Average mean and SD from these two measurements are shown here. Ther rNTP interference removal modification was not applied for these measurements.

Supplementary Table 5. Efficiency of detection reaction (%)

	dTTP	dATP	dCTP	dGTP	Extract volume per initial tissue weight
Liver	100	94	95	90	3 µl / mg
Liver	93	96	96	91	4 µl / mg
Heart	96	101	93		4 µl / mg
Heart				95	28 µl / mg*
Skeletal muscle	99	88	93	89	4 µl / mg

The reaction efficiencies were estimated by performing the measurements with and without a spike-in of purified dNTPs (corresponding to 0.25 pmol / reaction).
* Heart extracts had to be diluted further for the measurement of dGTP to reach the quantitative range of the assay. The 197-nt templates were used for these measurements

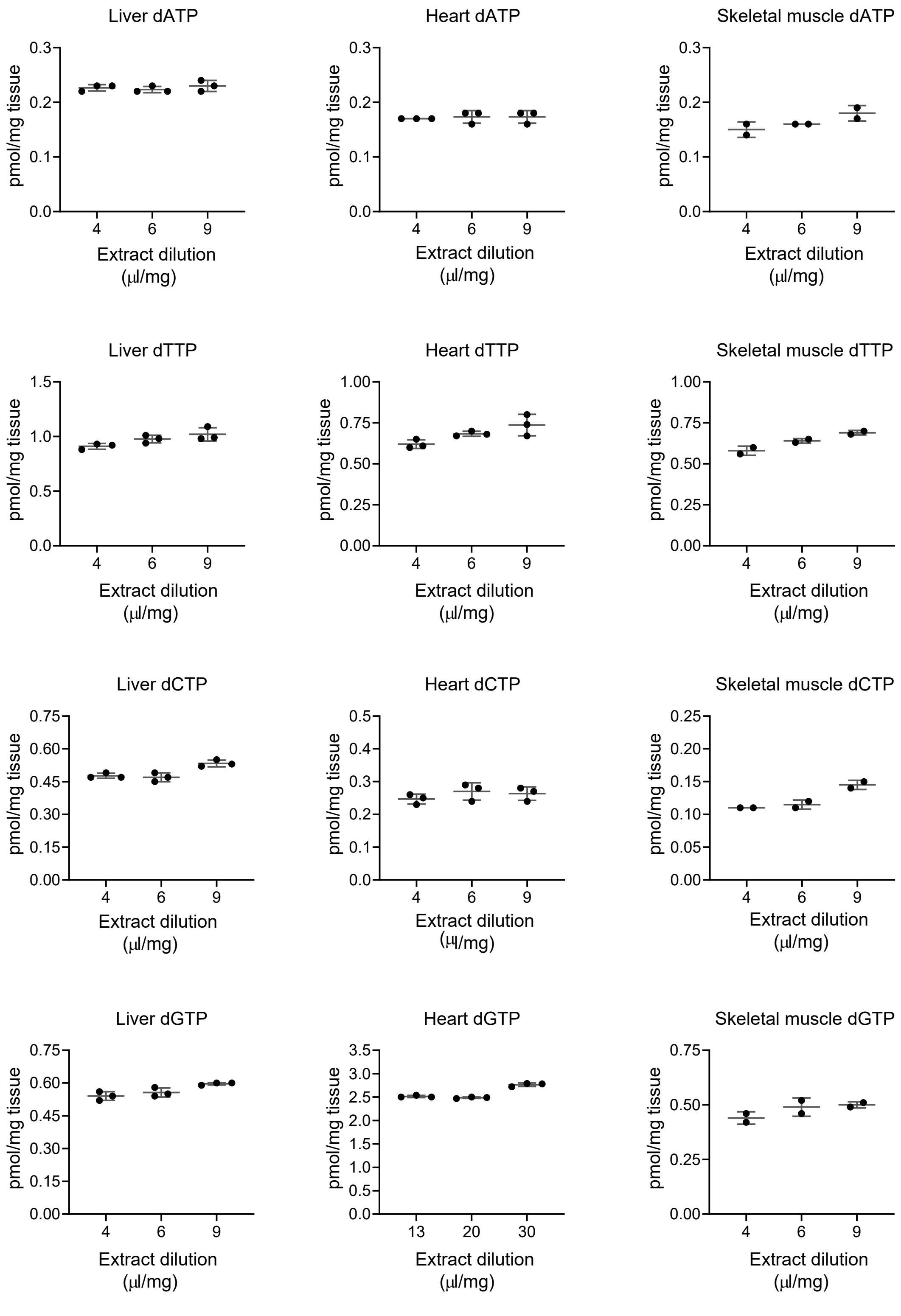

Supplementary Figure 7. Effect of tissue extract dilution on measured dNTP values. Three different extract dilutions (μl/mg initial tissue weight) were used for the measurements. The extract volume was half of the reaction volume. The 50-nt template reaction set up with RNAse HII was used for these measurements. The data points represent technical replicates from the same extract. The length of the error bars is two standard deviations.

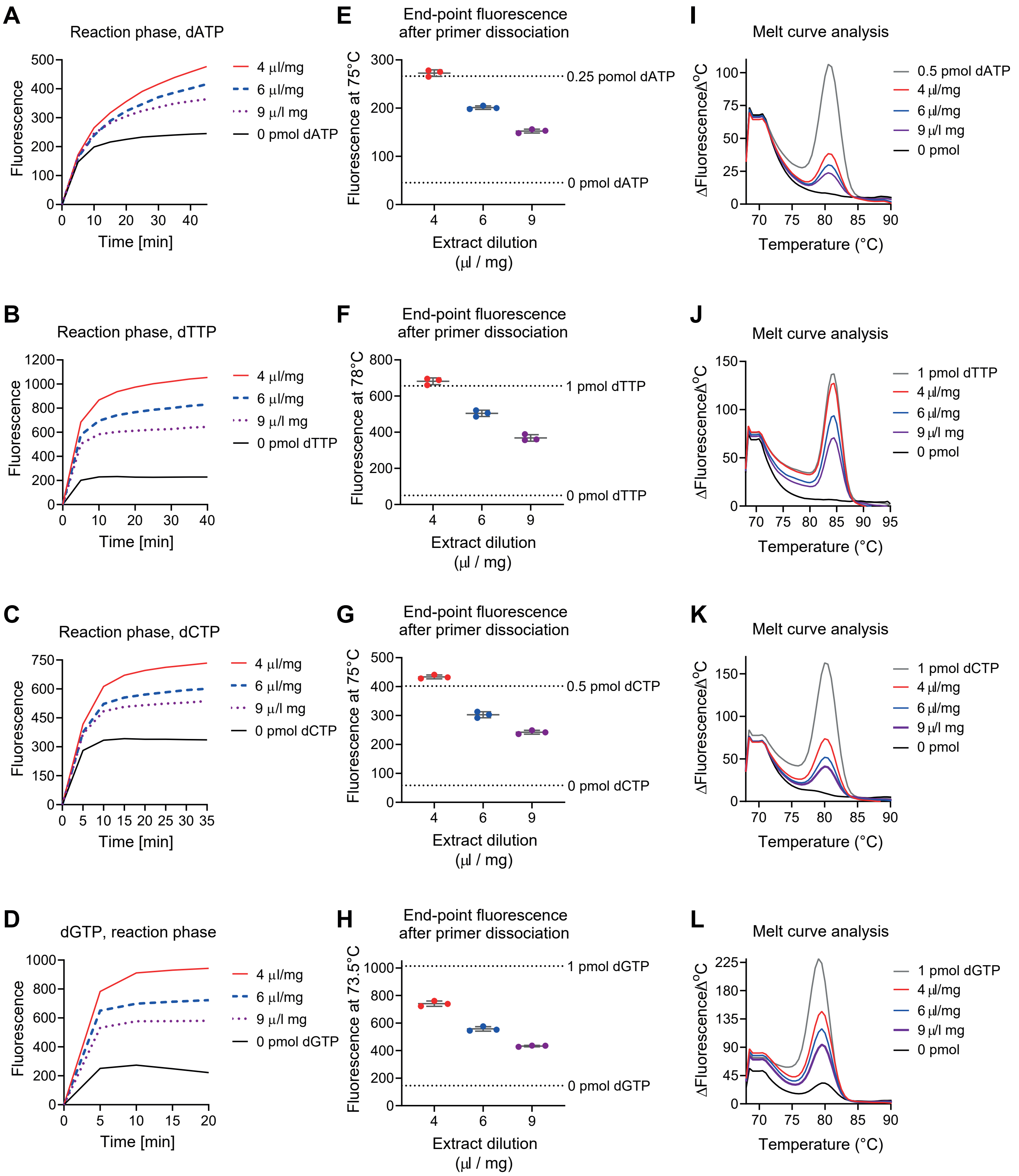

Supplementary Figure 8. Representative assay performance with liver extracts using 50-nt templates and thermostable RNAse HII. Three different extract dilutions (µl/mg initial tissue weight) were tested. The measurements were performed in technical triplicates. (**A-D**) Increase in fluorescence during the reaction phase at 66˚C. Initial fluorescence at this temperature was set as a baseline. (**E-H**) End-point fluorescence after raise of temperature to dissociate partially extended primers and RNAse HII-nicked products. The initial fluorescence at the same temperature before the reaction phase was set as the baseline. (**I-L**) Melt curve analysis of the reaction products. The peaks between 79 to 84ºC indicate a full-length product. The peak at approximately at 70ºC derives from the partially extended primer.

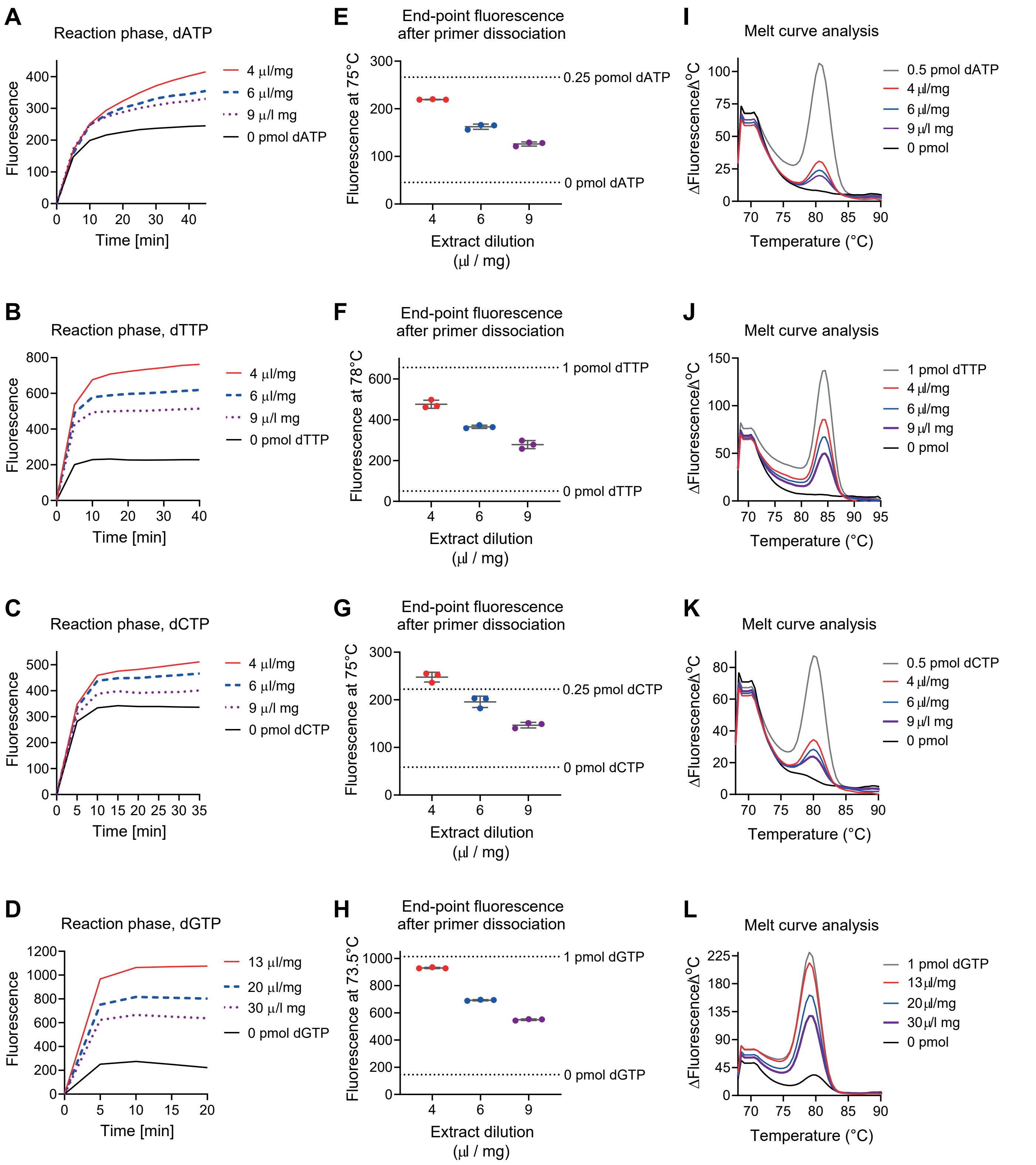

Supplementary Figure 9. Representative assay performance with heart extracts using 50-nt templates and thermostable RNAse HII. Three different extract dilutions (µl/mg initial tissue weight) were tested. The measurements were performed in technical triplicates. (**A-D**) Increase in fluorescence during the reaction phase at 66˚C. Initial fluorescence at this temperature was set as a baseline. (**E-H**) End-point fluorescence after elevation of temperature to dissociate partially extended primers and RNAse HII-nicked products. The initial fluorescence at the same temperature before the reaction phase was set as the baseline. (**I-L**) Melt curve analysis of the reaction products. The peaks between 79 to 84°C indicate a full-length product. The peak at approximately at 70°C derives from the partially extended primer.

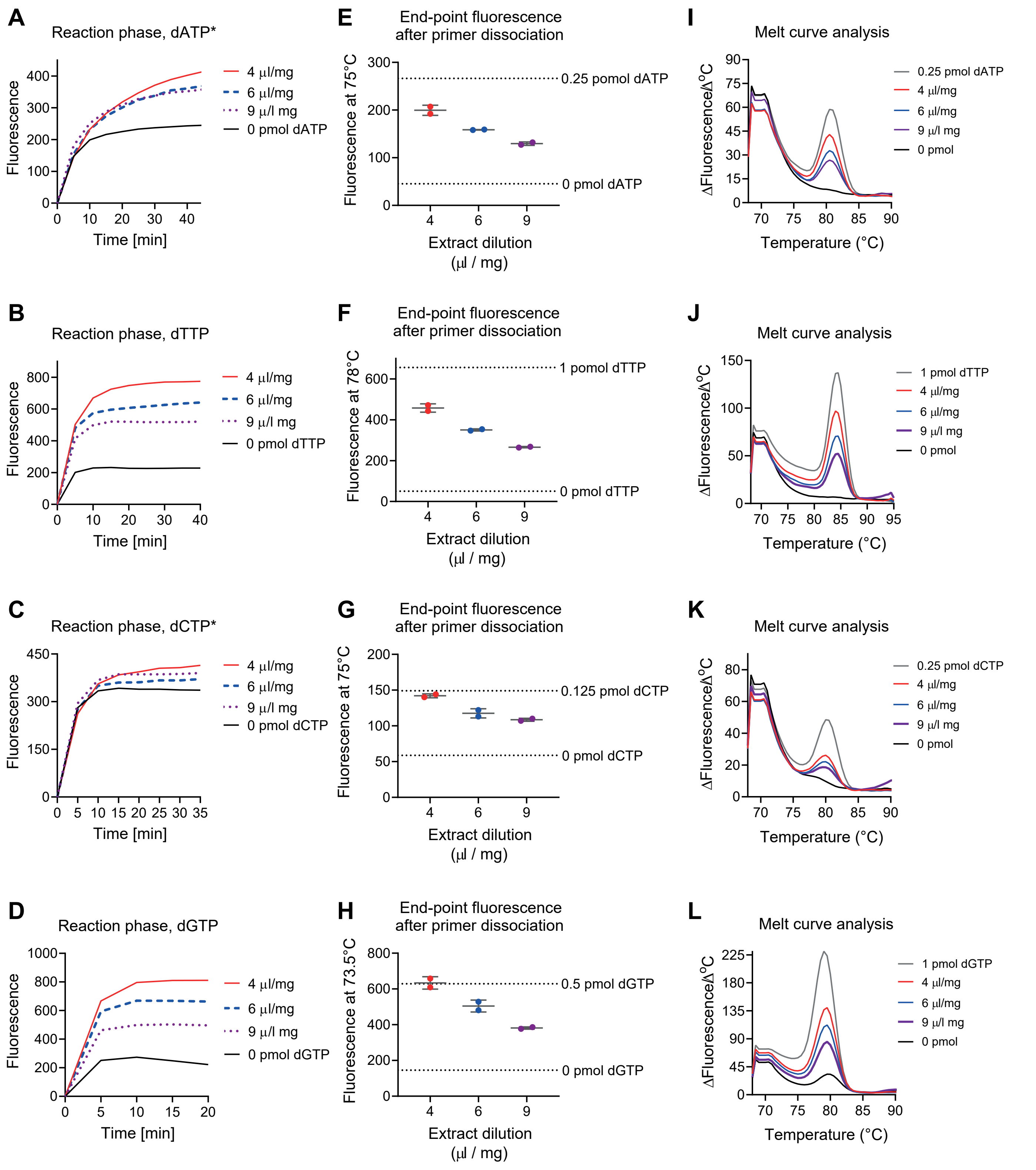

Supplementary Figure 10. Representative assay performance with skeletal muscle extracts using 50-nt templates and thermostable RNAse HII. Three different extract dilutions (µl/mg initial tissue weight) were tested. The measurements were performed in technical duplicates. (**A-D**) Increase in fluorescence during the reaction phase at 66˚C. Initial fluorescence at this temperature was set as a baseline. (**E-H**) End-point fluorescence after elevation of temperature to dissociate partially extended primers and RNAse HII-nicked products. The initial fluorescence at the same temperature before the reaction phase was set as the baseline. (**I-L**) Melt curve analysis of the reaction products. The peaks between 79 to 84ºC indicate a full-length product. The peak at approximately at 70ºC derives from the partially extended primer.
